# Chemical Constituents of the Bulbs of *Scilla peruviana* and Their Pancreatic Lipase Inhibitory Activity

**DOI:** 10.3390/ijms22031262

**Published:** 2021-01-27

**Authors:** Yukiko Matsuo, Asuka Yamashiro, Kanae Ootomo, Mika Nakagawa, Hiroko Tsuchihashi, Niro Inaba, Yoshihiro Mimaki

**Affiliations:** School of Pharmacy, Tokyo University of Pharmacy and Life Sciences, 1432-1, Horinouchi, Hachioji, Tokyo 192-0392, Japan; y104219@toyaku.ac.jp (A.Y.); y114037@toyaku.ac.jp (K.O.); y134172@toyaku.ac.jp (M.N.); 154150@toyaku.ac.jp (H.T.); ninaba@toyaku.ac.jp (N.I.); mimakiy@toyaku.ac.jp (Y.M.)

**Keywords:** *Scilla peruviana*, Asparagaceae, triterpene, lanosterol glycoside, homoisoflavanone, pancreatic lipase

## Abstract

*Scilla* species are used as medicinal plants and contain lanosterol-type triterpene glycosides. The phytochemical investigation of the bulbs of *Scilla peruviana* led to the isolation of 17 compounds, including three new rearranged pentacyclic-lanosterol glycosides (**1**–**3**) and two new homoisoflavanone glycosides (**12** and **13**). The structures of the undescribed compounds were determined by extensive spectroscopic analyses, including two-dimensional (2D) NMR. Among the triterpene glycosides, **2**, **3**, and **6** showed significant pancreatic lipase inhibitory activity in a concentration-dependent manner in vitro. The oral administration of scillascilloside D-2 (6) reduced serum triglyceride levels in a dose-dependent manner in soybean oil-loaded mice.

## 1. Introduction

*Scilla*, *Eucomis*, *Chionodoxa*, and *Muscari* species belong to the Asparagaceae (formerly Liliaceae) family and are rich sources of lanosterol-type triterpene glycosides [1,2,3,4]. Although the bulbs of *Scilla scilloides* (Lindl.) Druces, which contain homoisoflavones and triterpenes, has been used as a traditional medicine for the treatment of dermal inflammation [5,6]. *S. peruviana* L. are widely used as an ornamental plant. Previously, we conducted phytochemical examinations of the bulbs of *S. peruviana* and characterized two rearranged pentacyclic-lanosterol glycosides, peruvianosides A and B [7,8], a pentacyclic-norlanosterol glycoside, scillascilloside E-1 [9], and four hexacyclic-lanosterol glycosides with a modified spirocyclic side chain, including scillascilloside D-2 and scillasaponin B [10,11]. Peruvianoside A and scillasaponin B were found to show cyclic adenosine monophosphatase (cAMP) phosphodiesterase inhibitory activity [10,11]. We performed further phytochemical analyses of the bulbs of *S. peruviana* that resulted in the isolation of 17 compounds (**1**–**17**), including three undescribed rearranged pentacyclic-lanosterol glycosides (**1**–**3**) and two undescribed homoisoflavanone glycosides (**12** and **13**). The structures of the undescribed compounds were determined by extensive spectroscopic analyses, including two-dimensional (2D) NMR. Recently, some triterpenes and triterpene glycosides have been reported to exhibit potent pancreatic lipase inhibitory activity and reduce serum triglyceride (TG) levels in mice via pancreatic lipase inhibition, such as oleanane-type triterpene bisdesmosides (IC_50_ 81.4–223 μM). Pancreatic lipase plays a key role in the digestion of TG and is one of the targets for anti-obesity treatment [12,13,14]. Our previous study revealed that oral administration of cymbopogonol, a lupine-type triterpene, from *Cymbopogon citratus* reduced serum TG levels in mice via pancreatic lipase inhibitory activity [15]. On the other hand, homoisoflavanones have not been reported previously as pancreatic lipase inhibitors in natural products polyphenols [16]. In this study, we evaluated the pancreatic lipase inhibitory activity of the isolated lanosterol-type triterpene glycosides (**1**–**11**) and the effect of **6** on serum TG levels in soybean oil-loaded mice. 

## 2. Results and Discussion

### 2.1. Structural Elucidation

**Figure 1:**
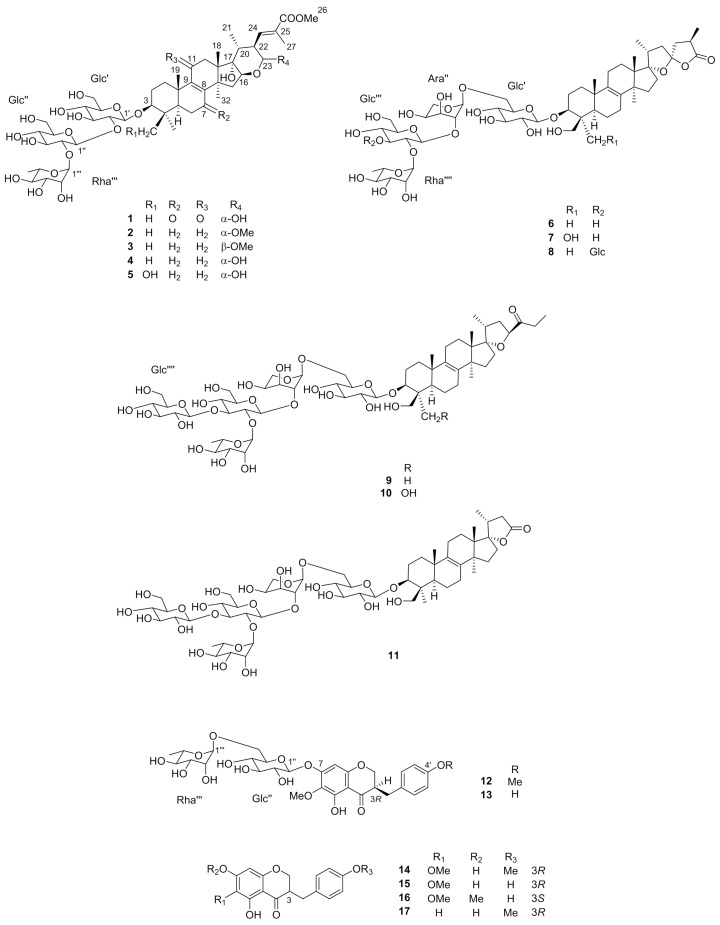
Isolated compounds from *Scilla peruviana*.

The bulbs of *S. peruviana* (11.2 kg dry weight) were treated with MeOH. After removing the solvent, the MeOH extract (981 g) was applied to a porous-polymer polystyrene resin (Diaion HP-20) column. The MeOH-eluted fraction was passed through column chromatography (CC) on silica gel and octadecylsilanized (ODS) silica gel, producing **4**–**11** and **14**–**17**. Compounds **4**–**11** and **14**–**17** were identified as peruvianoside A (**4**) [7], peruvianoside B (**5**) [7], (23*S*,25*R*)-3β,31-dihydroxy-17α,23-epoxy-5α-lanost-8-en-23,26-olactone3β-*O*-[*O*-α-l-rhamnopyranosyl-(1→2)-*O*-β-d-gluco-pyranosyl-(1→2)-*O*-α-l-arabinopyranosyl-(1→6)-*O*-β-d-glucopyranoside] (scillascilloside D-2) (**6**) [10], (23*S*,25*R*)-3β,30,31-trihydroxy-17α,23-epoxy-5α-lanost-5-en-23,26-olactone3-*O*-[*O*-α-l-rhamno-pyranosyl-(1→2)-*O*-β-d-glucopyranosyl-(1→2)-*O*-α-l-arabinopyranosyl-(1→6)-*O*-β-d-glucopyranoside] (**7**) [10], (23*S*,25*R*)-3β,31-dihydroxy-17α,23-epoxy-5α-lanost-8-en-23,26-olactone 3β-*O*-[*O*-β-d-glucopyranosyl-(1→3)-*O*-[α-l-rhamnopyranosyl-(1→2)]-*O*-β-d-glucopyranosyl-(1→2)-*O*-α-l-arabinopyranosyl-(1→6)-*O*-β-d-glucopyranoside] (scillanoside L-2) (**8**) [17], 15-deoxy-eucosterol3β-[*O*-β-d-glucopyranosyl-(1→3)-*O*-[α-l-rhamnopyranosyl-(1→2)]-β-d-glucopyranosyl-(1→2)-*O*-α-l-arabinopyranosyl-(1→6)-*O*-β-d-glucopyranoside] (scillascilloside E-1) (**9**) [9], 15-deoxy-30-hydroxyeucosterol3β-[*O-*β-d-glucopyranosyl-(1→3)-*O*-[α-l-rhamnopyranosyl-(1→2)]-β-d-glucopyranosyl-(1→2)-*O*-α-l-arabinopyranosyl-(1→6)-*O*-β-d-glucopyranoside] (scillanoside L-1) (**10**) [17], scillanostaside G (**11**) [18], (3*R*)-5,7-dihydroxy-6-methoxy-3-(4′-methoxybenzyl)-chroman-4-one (**14**) [19], (3*R*)-5,7-dihydroxy-3-(4′-hydroxybenzyl)-6-methoxy-chroman-4-one (**15**) [20], and (3*S*)-6,7-dimethoxy-5-hydroxy-3-(4′-hydroxybenzyl)-chroman-4-one (**16**) [21], and (3*R*)-5,7-dihydroxy-3-(4′-methoxybenzyl)-chroman-4-one (**17**) [22] (Figure 1). This is the first report on the isolation of **8**, **10**, **11**, and **14**–**17** from *S. peruviana*.

**Figure 2:**
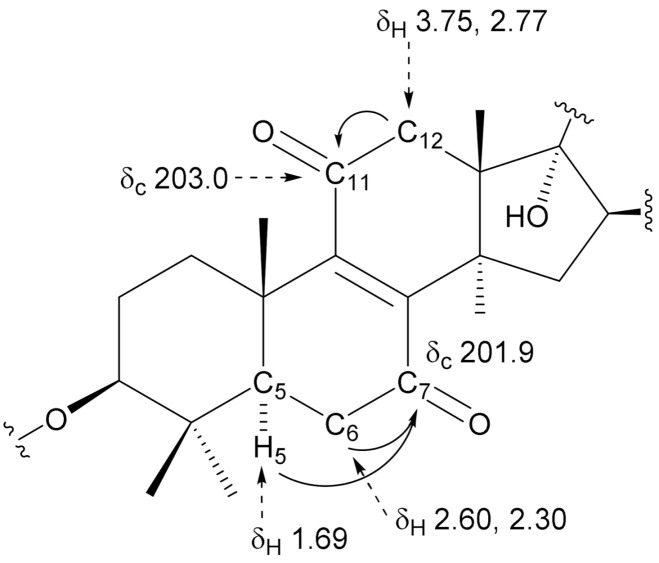
Key HMBC correlations of the aglycone moiety of **1**.

Compound **1** was obtained as an amorphous solid having the molecular formula C_49_H_74_O_22_, which was determined by high-resolution electrospray ionization time-of-flight mass spectrometry (HR-ESI-TOF-MS, *m/z* 1037.4563 [M + Na]^+^, calculated for C_49_H_74_NaO_22_: 1037.4569) and ^13^C NMR (49 carbon signals) data. The IR spectrum of **1** showed absorption bands of hydroxy groups at 3398 cm^−1^ and carbonyl groups at 1699, 1670, and 1587 cm^−1^. The ^1^H and ^13^C NMR spectra showed signals assignable to seven methyl groups [δ_H_ 1.99 (d, *J* = 1.2 Hz, Me-27), 1.84 (s, Me-32), 1.37 (s, Me-19), 1.30 (s, Me-30), 1.28 (s, Me-18), 1.16 (s, Me-31), and 1.07 (d, *J* = 6.9 Hz, Me-21); δ_C_ 30.7 (Me-32), 27.9 (Me-30), 21.1 (Me-18), 17.4 (Me-19), 16.5 (Me-31), 13.3 (Me-27), and 11.9 (Me-21)], two olefinic groups [δ_C_ 151.2 (C-8) and 151.6 (C-9) and δ_H_ 6.96 (dd, *J* = 10.6, 1.2 Hz, H-24); δ_C_ 143.5 (C-24) and 130.3 (C-25)], two oxymethine groups [δ_H_ 3.35 (dd, *J* = 11.8, 4.7 Hz, H-3); δ_C_ 88.4 (C-3), and δ_H_ 5.10 (dd, *J* = 7.4, 6.8 Hz, H-16); δ_C_ 79.4 (C-16)], an hemiacetalic proton and carbon [δ_H_ 5.24 (d, *J* = 6.5 Hz, H-23); δ_C_ 97.6 (C-23)], a tertiary carbon bearing a hydroxy group [δ_C_ 82.1 (C-17)], a methyl ester group [δ_C_ 168.3 (C-26) and δ_H_ 3.71(3H, s); δ_C_ 51.6], and three anomeric protons and carbons [δ_H_ 6.38 (br s, H-1′′′), 5.83 (d, *J* = 6.9 Hz, H-1′′), and 4.91 (d, *J* = 7.7 Hz, H-1′); δ_C_ 105.2 (C-1′), 102.1 (C-1′′′), and 101.9 (C-1′′)] (Table 1 and Table 2). These ^1^H and ^13^C NMR spectral features of **1** were similar to those of **4 [5]**, except for the signals arising from the B and C-rings parts of the aglycone moiety. The two methylene signals at δ_C_ 26.5 (C-7) and 21.1 (C-11) observed in the ^13^C NMR spectrum of **4** were displaced by two conjugated carbonyl signals at δ_C_ 201.9 (C-7) and 203.0 (C-11) in **1**. Furthermore, an olefinic carbon signals of **4** at δ_C_ 135.9 (C-8) and 134.6 (C-9) were shifted downfield by 15.3 and 17.0 ppm and observed at δ_C_ 151.2 (C-8) and 151.6 (C-9) in **1**, respectively. These data indicated that **1** was a 7,11-dioxo derivate of **4**. This was confirmed by long-range correlations between H_2_-12 at δ_H_ 3.75 and 2.77 and C-11 at δ_C_ 203.0, and between H-5 at δ_H_ 1.69/H_2_-6 at δ_H_ 2.60 and 2.30 and C-7 at δ_C_ 201.9 in the heteronuclear multiple bond correlation (HMBC) spectrum of **1** (Figure 2). The configuration of the C-23 hydroxy group was confirmed to be α based on the spin-coupling constants of ^3^*J*_H-20,H-22_ = 11.8 Hz, ^3^*J*_H-22,H-23_ = 6.5 Hz, and ^3^*J*_H-22,H-24_ = 10.6 Hz, and NOE correlations between H-23 and H-24/H-20 [10]. Enzymatic hydrolysis of **1** gave d-glucose (Glc) and l-rhamnose (Rha), which were identified by comparison of their refractive index and optical rotations with those of authentic samples using HPLC. The anomeric conformation of the Glc and Rha groups was ascertained by the *J* values of their anomeric protons, respectively. In the HMBC spectrum of **1**, long-range correlations were observed between H-1′′′ of Rha at δ_H_ 6.38 and C-2′′ of Glc at δ_C_ 78.7, H-1′′ of Glc at δ_H_ 5.83 and C-2′ of Glc at δ_C_ 78.7, and between H-1′ of Glc at δ_H_ 4.91 and C-3 of the aglycone at δ_C_ 88.4. Accordingly, **1** was determined to be 7,11-dioxo-peruvianoside A.

The ^1^H and ^13^C NMR spectral features of **2** (C_50_H_80_O_20_) and **3** (C_50_H_80_O_20_) were closely related to those of **4**. However, when the ^13^C NMR spectra of **2** and **3** were compared with that of **4**, the signal assignable to the hemiacetal carbon at δ_C_ 97.7 (C-23) in **4** was shifted downfield by 12.7 ppm in **2** and 6.2 ppm in **3**, and observed at δ_C_ 110.4 in **2** and at δ_C_ 103.9 in **3**. The ^1^H NMR spectra of **2** and **3** displayed a deshielded methyl singlet signal at δ_H_ 3.49 and δ_H_ 3.42, respectively (Table 1). In the HMBC spectrum of **2**, the methyl singlet at δ_H_ 3.49 showed a long-range correlation with C-23 at δ_C_ 110.4. Additionally, the methyl singlet at δ_H_ 3.42 exhibited an HMBC correlation with C-23 at δ_C_ 103.9 in **3**. These data indicated that the hemiacetal moiety of **4** was changed to the methyl acetal in **2** and **3**. In the NOESY spectrum of **2**, NOE correlations between H-23 and H-24 and between H-22 and C_23_-OMe/Me-27, and a spin-coupling constant of ^3^*J*_H-22,H-23_ = 6.3 Hz showed the configuration at C-23 to be α in **2**. On the other hand, NOE correlations between C_23_-OMe and H-20/H-24 and between H-23 and H-22/Me-27, and a spin-coupling constant of ^3^*J*_H-22,H-23_ = 5.8 Hz gave evidence that **3** was the C-23 epimer of **2**. The triglycoside moiety attached to C-3 of the aglycone of **2** and **3** was ascertained to be the same as that of **4** by the analysis of the HMBC spectra of **2** and **3**. Therefore, **2** and **3** were identified as 23α-methyl-peruvianoside A and 23β-methyl-peruvianoside A. The ^1^H NMR spectrum of **12** (C_30_H_38_O_15_) was essentially analogous to that of the homoisoflavanone (**14**), showing signals for two methylene groups at δ_H_ 4.36 (dd, *J* = 11.5, 4.0 Hz, H-2a) and 4.19 (dd, *J* = 11.5, 6.5 Hz, H-2b), and δ_H_ 3.13 (dd, *J* = 14.0, 9.0 Hz, H-9a) and 2.72 (dd, *J* = 14.0, 7.0 Hz, H-9b), a methine proton at δ_H_ 2.96 (m, H-3), 1,4-disubstituted aromatic ring at δ_H_ 7.16 (d, *J* = 8.5 Hz, H-2′ and H-6′) and 6.86 (d, *J* = 8.5 Hz, H-3′ and H-5′), and an aromatic proton at δ_H_ 6.31 (s, H-8), and two methoxy groups at δ_H_ 3.77 (s) and 3.75 (s) (Table 3). Furthermore, signals for two anomeric protons and carbons were observed at δ_H_ 4.96 (d, *J* = 7.5 Hz); δ_C_ 101.7 and δ_H_ 4.68 (br s); δ_C_ 102.2. The enzymatic hydrolysis of **12** gave 5,7-dihydroxy-6-methoxy-3-(4′-methoxybenzyl)-chroman-4-one (**12a**) [19] and d-glucose (Glc) and l-rhamnose (Rha) as the carbohydrate moieties. The absolute configuration at C-3 was confirmed to be *R* because of the CD spectrum [(MeOH, ∆ε): 284 (−1.98) nm], and the specific rotation of **12a** almost agreed with that of **14**. In the HMBC spectrum of **12**, long-range correlations were observed between the anomeric proton (H-1′′′) of Rha at δ_H_ 4.68 and C-6′′ of Glc at δ_C_ 67.6, and between H-1′′ of Glc at δ_H_ 4.96 and C-7 of the aglycone at δ_C_ 160.8. Thus, **12** was identified as (3*R*)-5-hydroxy-6-methoxy-3-(4′-methoxybenzyl)-7-[(*O*-α-l-rhamnopyranosyl-(1→6)-β-d-glucopyranosyl)oxy]-chroman-4-one.

The ^1^H and ^13^C NMR spectra of **13** (C_29_H_36_O_15_) showed a close similarity to those of **12**, including the signals attributed to the sugar moiety attached to C-7. However, **13** differed from **12** in the lack of the ^1^H and ^13^C signals for the methoxy group at C-4′ [δ_H_ 3.77 (s); δ_C_ 55.7] (Table 3). Furthermore, the C-4′ signal of **13** was shifted upfield by 1.7 ppm when compared with that of **12**. These data implied that the aglycone of **13** corresponded to **15** and that **13** had an *O*-α-l-rhamnopyranosyl-(1→6)-β-d-glucopyranosyl group at C-7. Thus, **13** was identified as (3*R*)-5-hydroxy-6-methoxy-3-(4′-hydroxybenzyl)-7-[(*O*-α-l-rhamnopyranosyl-(1→6)-β-d-glucopyranosyl)oxy]-chroman-4-one.

Structures of known compounds **14**–**17**, including their absolute configurations, were determined based on a spectroscopic analysis [19,20,21,22].

### 2.2. Lipase Inhibitory Activity

**Figure 3:**
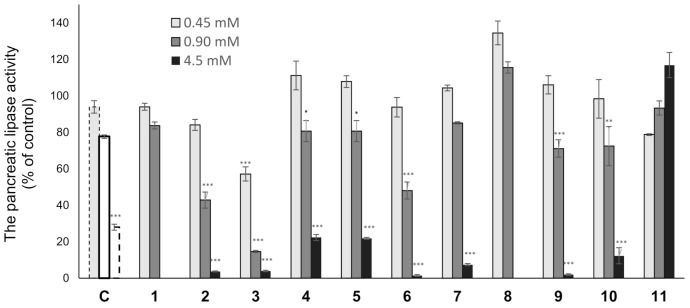
The pancreatic lipase activity of **1**–**11.** Compounds **1** and **8** (0.45 and 0.90 mM), **2**–**7** and **9**–**11** (0.45, 0.90, and 4.5 mM), and cetilistat (c) (0.1 [ ], 1.0 [ ], and 10 [ ] μM); Data are represented as the mean ± S.E.M. of three experiments performed in triplicate; *** *p* < 0.0001 vs. control, ** *p* < 0.001 vs. control, **˙** *p* < 0.05 vs. control.

**Figure 4:**
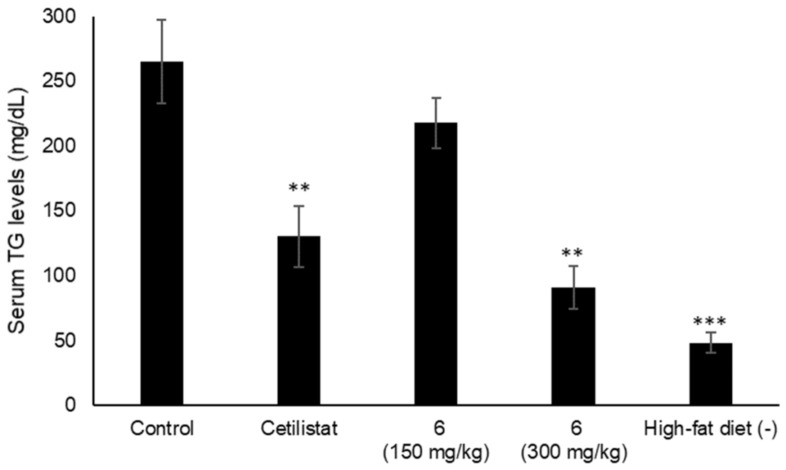
Effects of **6** on the serum TG levels in mice fed a single-dose administration of a high-fat diet. Compound 6 (150 or 300 mg/kg) and cetilistat (180 mg/kg); Data are represented as the mean ± S.E.M. (*n* = 5); *** *p* < 0.0001 vs. control, ** *p* < 0.001 vs. control.

Various types of triterpenes and triterpene glycosides have been reported as pancreatic lipase inhibitors and expected to be promising treatments for obesity [12,13,14,16,23]. The pancreatic lipase inhibitory activity of the isolated lanosterol-type triterpene glycosides (**1**–**11**) was measured using a commercially available kit (Figure 3 and Table 4). Cetilistat was used as a positive control (IC_50_ 1.43 ± 0.052 μM). As a result, **2**, **3**, and **6** showed the inhibitory activity in a dose-dependent manner (IC_50_ 0.84 ± 0.029, 0.59 ± 0.020, and 0.81 ± 0.029 mM). Interestingly, **11**, a tetranorlanosterol glycoside, inhibited the pancreatic lipase activity in an inverted dose-dependent manner. Of the compounds with the peruvianoside A derivatives (**1**–**5**), the modification of the hemiacetal group to the corresponding methyl acetal group (**2** and **3**) enhanced the lipase inhibitory activity. In the lanosterol derivatives with a spiro-lactone group (**6**–**8**), **6** had the most potent lipase inhibitory activity. Next, the effects of **6** on TG levels in ddY mice fed a single-dose administration of a high-fat diet (intralipid) were examined (Figure 4). Compound **6** at 150 or 300 mg/kg was orally administrated to mice, respectively, 15 min before intralipid loading. Serum TG levels were determined at 120 min after intralipid oral treatment. In the control group, serum TG levels increased from 48.3 ± 8.2 to 264.9 ± 32.2 mg/dL following administration of intralipid compared to those of the untreated group. When the mice were treated with cetilistat as a positive control, serum TG levels were held at 130.2 ± 23.4 mg/dL. Although inhibition potency of cetilistat in vivo was weaker than that of the lipase inhibitory activity in vitro (IC_50_ 1.43 μM), the same range was reported in previous works [24,25]. The single oral administration of **6** at 300 mg/kg significantly suppressed the elevation in serum TG levels, with values held at 90.9 ± 16.2 mg/dL in mice.

3β-Hydroxylanosta 9,24-dien-21-oic acid, a lanostane-type triterpene, isolated from *Protorhus longifolia*, was reported to inhibit the pancreatic lipase activity [26]. However, no previous reports have shown the effect of purified lanostane-type triterpene glycoside on both lipase inhibition in vitro and TG reduction in vivo [27]. This is the first report of the lipase inhibitory activity of lanosterol glycosides. The inhibition of lipase activity might be only one of the possible mechanisms inhibiting triglycerides absorption.

In conclusion, five rearranged pentacyclic-lanosterol glycosides (**1**–**5**), three hexacyclic-lanosterol glycosides with a modified spirocyclic side chain (**6**–**8**), two pentacyclic-norlanosterol glycosides (**9** and **10**), a pentacyclic-tetranorlanosterol glycoside (**11**), a homoisoflavanone glycosides (**12** and **13**), and four homoisoflavanones (**14**–**17**) were isolated from the MeOH extract of *S. peruviana* bulbs. Their structures were determined by extensive spectroscopic analysis and the results of hydrolytic cleavage. Compounds **1**–**3**, **12**, and **13** are undescribed glycosides, and **8**, **10**, **11**, and **14**–**17** were isolated from *S. peruviana* for the first time. The pancreatic lipase inhibitory activity of the isolated triterpene glycosides (**1**–**11**) was evaluated. Compounds **2**, **3**, and **6** showed significant lipase inhibitory activity in a dose-dependent manner (IC_50_ 0.84 ± 0.029, 0.59 ± 0.020, and 0.81 ± 0.029 mM) and a single oral dose of **6** at 300 mg/kg reduced serum TG levels in mice loaded with intralipid. It is concluded that **6** suppress elevated serum TG levels partially via lipase inhibition. Further studies are needed to reveal the mechanism of the effects of **6** on the serum TG reduction.

## 3. Materials and Methods

### 3.1. General Experimental Procedures

The instruments and experimental conditions were the same as those described in a previous paper [28]. Optical rotations were measured on a JASCO P-1030 automatic digital polarimeter (Jasco). IR spectra were obtained using a JASCO FT-IR 620 spectrophotometer (Jasco). NMR spectra were recorded on a Bruker DRX-500 or AV-600 spectrometer (Bruker) using standard Bruker pulse programs. Chemical shifts (δ) were given with reference to tetramethylsilane (TMS) as an internal standard. HR-ESI-TOF-MS data were obtained using a Water-Micromass LCT mass spectrometer (Waters-Micromass). CC was conducted by Diaion HP-20 (Mitsubishi-chemical), silica gel Chromatorex BW-300 (Fuji-Silysia Chemical), and ODS silica gel COSMOSIL 75C_18_-OPN (Nacalai Tesque). Analytical TLC was performed on precoated silica gel 60 F_254_ or RP18 F_254_S plates (0.25 mm thick) (Merck). The spots were detected by spraying the plates with 10% H_2_SO_4_ aqueous solution and then heating. HPLC was conducted using a system consisting of a CCPM pump (Shimadzu), an RI-8021 (Tosoh) or a Shodex OR-2 (Showa-Denko) detector, and a Rheodyne injection port (Rohnert Park). A TSK gel ODS-100Z column (10 mm i.d. × 250 mm, 5 µm) (Tosoh) was used for the preparative HPLC. Enzymatic hydrolysis was carried out using naringinase (EC 232-962-4, Sigma), acetic acid (AcOH) and potassium acetate (AcOK) (Wako).

### 3.2. Plant Material

The bulbs of *Scilla peruviana*. (11.2 kg dry weight) were obtained from Takii (Kyoto, Japan) in 2014. The bulbs were cultivated, and the morphological characteristics of the flowering plants allowed us to identify the plant material. A voucher specimen was deposited at the Herbarium of the Tokyo University of Pharmacy and Life Sciences (KS-2014-017).

### 3.3. Extraction and Isolation

The bulbs of *S. peruviana* (11.2 kg) were treated with MeOH (50 L) at 50 °C for 2 h. After evaporating the solvent in vacuo, the MeOH extract (981 g) was subjected to a Diaion HP-20 column (2200 g, 85 mm i.d. × 600 mm) and successively partitioned by eluting with MeOH–H_2_O (3:7; 1:1), MeOH, EtOH, and EtOAc (each 10 L) in order of decreasing polarity. The MeOH-eluted fraction (221 g) was passed through a silica gel CC (2400 g, 42 mm i.d. × 430 mm) eluted with gradient mixtures of CHCl_3_–MeOH–H_2_O (20:10:1; 7:4:1) and MeOH, to give 8 fractions (Frs. A-H). Fr. A was separated by ODS silica gel CC (930 g, 32 mm i.d. × 300 mm) eluted with MeOH–H_2_O (2:1) and silica gel CC (580 g, 22 mm i.d. × 380 mm) eluted with CHCl_3_–MeOH–H_2_O (40:10:1) to give **13** (13.1 mg). Fr. C was separated by silica gel CC (1800 g, 80 mm i.d. × 400 mm) eluted with EtOAc–MeOH (2:1; 1:1) and sequentially ODS silica gel CC (700 g, 30 mm i.d. × 250 mm) eluted with MeOH–H_2_O (2:1; 3:1) and MeCN–H_2_O (1:2; 2:3) to yield **7** (22.1 mg). Fr. E was fractionated by ODS silica gel CC (930 g, 35 mm i.d. × 300 mm) using MeOH–H_2_O (2:1; 3:1; 4:1) and MeCN–H_2_O (1:2; 2:3; 1:1), and preparative TLC using CHCl_3_–MeOH–H_2_O (20:10:1) to give **1** (15.5 mg), **4** (16.0 mg), and **10** (43.8 mg). Fr. E-1 was separated by ODS silica gel CC (930 g, 35 mm i.d. × 300 mm) eluted with MeOH–H_2_O (2:1; 3:1; 4:1) and silica gel CC (430 g, 22 mm i.d. × 280 mm) eluted with CHCl_3_–MeOH–H_2_O (70:40:1; 10:10:1) to give **12** (21.8 mg). Fr. F was purified by ODS silica gel CC (930 g, 32 mm i.d. × 320 mm) using MeOH–H_2_O (2:1; 3:1; 4:1) and MeCN–H_2_O (1:2; 2:3; 1:1), and preparative HPLC using MeCN–H_2_O (2:3) to yield **5** (16.8 mg), **6** (59.0 mg) and **8** (15.6 mg). Fr. G was fractionated by ODS silica gel CC (500 g, 22 mm i.d. × 330 mm) using MeCN–H_2_O (1:2; 2:3) and silica gel CC (450 g, 22 mm i.d. × 280 mm) eluted with CHCl_3_–MeOH–H_2_O (10:10:1) to give **2** (69.3 mg), **3** (56.1 mg), **9** (25.0 mg) and **11** (50.8 mg). The EtOH-eluted fraction (5.4 g) passed through a silica gel CC (1000 g, 32 mm i.d. × 320 mm) eluted with gradient mixtures of hexane–EtOAc (3:1; 1:1) and MeOH, to give 7 fractions (Frs. a–g). Fr. d was fractionated by ODS silica gel CC (280 g, 17 mm i.d. × 300 mm) using MeOH–H_2_O (5:2; 7:1) and preparative HPLC using MeOH–H_2_O (4:1) to give **14** (7.4 mg), **15** (42.3 mg), **16** (74.2 mg), and **17** (9.7 mg).

### 3.4. Structural Characterization

Compound **1**: An amorphous solid; [α]_D_^25^ −10.1 (*c* 0.27, MeOH); IR ν_max_ (film) cm^−1^: 3398 (OH), 2963 (CH), 1699 (C=O ester), 1670 (C=O ketone and C=C), 1587 (C=O ketone); UV λ_max_ (MeOH) nm (log ε): 264 (3.98) (C=O ketone), 204 (4.26) (C=O ester). ^1^H and ^13^C NMR (500 and 125 MHz, C_5_D_5_N), see Table 1 and Table 2; HR-ESI-TOF-MS (*m/z*: 1037.4563 [M + Na]^+^, calculated for C_49_H_74_NaO_22_: 1037.4569).

Enzymatic hydrolysis of **1**: Compound **1** (4.9 mg) was treated with naringinase (80 mg) in AcOH/AcOK buffer (pH 4.3, 4.0 mL) for 24 h. The crude hydrolysate was subjected to Diaion HP-20 CC (6 mm i.d. × 200 mm) eluted with MeOH–H_2_O (3:7), Me_2_CO–EtOH (1:1), and MeOH to yield the sugar fraction (0.31 mg). The aglycone moiety of **1** was decomposed. The sugar fraction of **1** was analyzed by HPLC under the following conditions: Capcell Pak NH_2_ UG80 column (4.6 mm i.d.× 250 mm, 5 μm, Shiseido), mobile phase of MeCN–H_2_O (7:3), detection by refractive index and optical rotation, and flow rate of 1.0 mL/min. d-Glucose and l-rhamnose were identified by comparing their retention times and optical rotation with those of authentic samples. *t*_R_ (min): 16.85 (d-glucose, positive optical rotation) and 7.97 (l-rhamnose, negative optical rotation).

Compound **2**: An amorphous solid; [α]_D_^25^ +10.4 (*c* 0.17, MeOH); IR ν_max_ (film) cm^−1^: 3390 (OH), 2935 (CH), 1699 (C=O ester), 1648 (C=C); UV λ_max_ (MeOH) nm (log ε): 219 (4.37) (C=C), 205 (4.28)(C=O ester); ^1^H and ^13^C NMR (500 and 125 MHz, C_5_D_5_N), see Table 1 and Table 2; HR-ESI-TOF-MS (*m/z*: 1023.5141 [M + Na]^+^, calculated for C_50_H_80_NaO_20_: 1023.5141).

Compound **3**: An amorphous solid; [α]_D_^25^ −31.1 (*c* 0.36, MeOH): IR ν_max_ (film) cm^−1^: 3382 (OH), 2927 (CH), 1715 (C=C), 1643 (C=O ester); UV λ_max_ (MeOH) nm (log ε): 220 (4.42) (C=C), 206 (4.42) (C=O ester); ^1^H and ^13^C NMR (500 and 125 MHz, C_5_D_5_N), see Table 1 and Table 2; HR-ESI-TOF-MS (*m/z*: 1023.5140 [M + Na]^+^, calculated for C_50_H_80_NaO_20_: 1023.5141).

Compound **12**: An amorphous solid; [α]_D_^25^ −13.1 (*c* 0.05, MeOH): IR ν_max_ (film) cm^−1^: 3413 (OH), 2922 (CH), 1730(C=O), 1590, 1456 (aromatic rings); UV λ_max_ (MeOH) nm (log ε): 284 (3.72), 205 (C=O ester); CD λ_max_ (MeOH) nm (∆ε): 282 (−1.80); ^1^H and ^13^C NMR (500 and 125 MHz, CD_3_OD), see Table 3; HR-ESI-TOF-MS (*m/z*: 661.2119 [M + Na]^+^, calculated for C_30_H_38_NaO_15_: 661.2108).

Enzymatic hydrolysis of **12**: Compound **12** (4.0 mg) was subjected to enzymatic hydrolysis with naringinase (80 mg) as described for **1** to give **12a** (2.0 mg) and a sugar fraction (1.2 mg). The HPLC analysis of the sugar fraction under the same conditions as in the case of **1** showed the presence of d-glucose and l-rhamnose. *t*_R_ (min): 12.71 (d-glucose, positive optical rotation) and 7.83 (l-rhamnose, negative optical rotation).

Compound **12a**: An amorphous solid; [α]_D_^25^ −32.8 (*c* 0.05, MeOH): CD λ_max_ (MeOH) nm (∆ε): 284 (−1.98); ^1^H NMR (500 MHz, CD_3_OD) δ_H_ 7.15 and 6.86 (each 2H, d, *J* = 8.2 Hz), 5.92 (s), 4.30 (dd, *J* = 11.4, 4.2 Hz), 4.15 (dd, *J* = 11.4, 7.0 Hz), 3.76 (s), 3.71 (s), 3.12 (dd, *J* = 13.8, 4.3 Hz), 2.83 (m), 2.70 (dd, *J* = 13.8, 10.5 Hz); HR-ESI-TOF-MS (*m/z*: 353.1005 [M + Na]^+^, calculated for C_18_H_18_NaO_6_: 353.1001).

Compound **13**: An amorphous solid; [α]_D_^25^ −1.2 (*c* 0.20, MeOH): IR ν_max_ (film) cm^−1^: 3411 (OH), 2924 (CH), 1740(C=O),1626, 1539 (aromatic rings); UV λ_max_ (MeOH) nm (log ε): 285 (3.64), 205 (C=O ester); CD λ_max_ (MeOH) nm (∆ε): 271 (−0.01); ^1^H and ^13^C NMR (500 and 125 MHz, CD_3_OD), see Table 3; HR-ESI-TOF-MS (*m/z*: 647.1954 [M + Na]^+^, calculated for C_29_H_36_NaO_15_: 647.1952).

### 3.5. Lipase-Inhibitory Activity

The pancreatic lipase activity of each test sample was measured by a modified method using a commercially available kit (Lipase Kit S, Dainippon Pharmaceutical). The purities of test samples were confirmed by HPLC analysis (Figure S6 in Supplementary Materials). Cetilistat was used as a positive control at 0.1, 1.0, and 10 μM. Test sample was dissolved in EtOH–H_2_O (1:1) and diluted to concentrations of 0.45, 0.90, and 4.5 mM, respectively. Lipase (lipase from porcine pancreas, TYPE II, 100–500 units/mg) was purchased from Sigma-Aldrich. Briefly, test sample was mixed with enzyme buffer, and incubated for 5 min at 30 °C. After incubation, 2,3-dimercapto-1-propanol tributyrate (BALB) as substrate was added and the enzyme reaction was proceeded for 30 min at 30 °C. Lipase activity was determined by measuring the absorbance of the resulting 5-thio-2-nitrobenzoate anion at 412 nm using a microplate reader. The inhibition rate was calculated by the following formula: Inhibition rate (%) = [1 − (Δ*A*_sample_ − Δ*A*_blank_)/(Δ*A*_control_ − Δ*A*_blank_)] × 100. The inhibitory concentration (IC_50_) was calculated by log-Probit analysis.

### 3.6. Animals

Male ddY mice, 5 weeks old, were purchased from Japan Laboratory Animals and maintained in the animal facility of the Tokyo University of Pharmacy and Life Sciences. The animals were housed in cages under controlled temperature (23 ± 2 °C) and a 12 h light-dark cycle. The mice were allowed free access to breeding food and water. All experiments were approved by the Tokyo University of Pharmacy and Life Sciences Animal Use Committee (approval number: P17-84, date of approval: 24 April 2017).

### 3.7. Measurement of Triglyceride in Animals

After 5 weeks, mice were fasted overnight and randomly divided into five groups (*n* = 5). The body weight of each mouse was measured (24.0–30.7 g/body). 0.5% Carboxymethyl cellulose (CMC; Wako Pure Chemical Industries), cetilistat (Tokyo Kasei, purity > 98.0%; 180 mg/kg), or compound **6** (150 or 300 mg/kg) were prepared for test samples and the intralipid (soybean oil 20% emulsion, 20 mL/kg, Sigma-Aldrich) was used for the high-fat diet. Single dose of 0.5% CMC, cetilistat, and compound **6** (150 or 300 mg/kg) were orally administered to mice in each group (*n* = 5) 15 min before the intralipid loading. Blood samples were collected from the abdominal vena cava 120 min after the intralipid-loaded treatment and centrifuged (700× *g* for 10 min) to obtain serum samples. The TG levels in serum were measured using a commercially available kit (Wako Triglyceride E-Test kit, Wako Pure Chemical Industries).

### 3.8. Statistical Analysis

Data are represented as the mean ± standard error of the mean (S.E.M.) of three experiments performed in triplicate. Dunnett’s test was used for statistical analysis, and the level of significance is indicated by *p* values. All statistical analyses were performed with R (R version 3.2.4).

## Figures and Tables

**Table 1:** ^1^H and ^13^C NMR (500 and 125 MHz, C_5_D_5_N) spectroscopic assignments of the aglycone moieties of **1**–**3**.

	1				2				3			
C	δ_H_		*J* (Hz)	δ_C_	δ_H_		*J* (Hz)	δ_C_	δ_H_		*J* (Hz)	δ_C_
1a	3.00	dt	13.8, 3.3	34.4	1.54	m		35.8	1.62	m		35.8
b	1.21	br dd	13.8, 3.4		1.10	m			1.19	m		
2a	2.28	m		26.9	2.26	m		27.1	2.26	m		27.1
b	1.92	m			1.85	m			1.84	m		
3	3.35	dd	11.8, 4.7	88.4	3.33	dd	11.3, 4.2	89.7	3.36	dd	11.7, 4.2	89.7
4	—			36.9	—			39.8	—			39.8
5	1.69	br d	7.8	50.7	1.15	br d	7.3	51.0	1.11	br d	7.8	51.1
6a	2.60	br d	8.7	36.5	1.70	dd	12.1, 6.2	18.4	1.71	dd	12.0, 6.1	18.4
b	2.30	m			1.51	m			1.50	m		
7	—			201.9	2.08	m		26.6	2.10	m		26.6
					1.51	m			1.39	m		
8	—			151.2	—			135.9	—			135.9
9	—			151.6	—			135.0	—			135.1
10	—			39.9	—			37.0	—			37.1
11	—			203.0	2.11	m		20.8	2.14	m		20.8
					2.00	m			2.06	m		
12a	3.75	d	15.9	48.9	2.45	dt	13.0, 9.2	25.4	2.45	m		25.7
b	2.77	d	15.9		1.58	br dd	13.0, 4.9		1.63	br dd	13.0, 3.4	
13	—			52.4	—			48.5	—			48.6
14	—			47.4	—			48.1	—			48.3
15a	3.36	dd	12.9, 7.4	42.5	2.22	m		44.4	2.23	dd	12.2, 7.8	44.4
b	2.50	dd	12.9, 6.8		2.03	m			2.03	m		
16	5.10	dd	7.4, 6.8	79.4	5.06	dd	9.2, 6.2	80.2	4.52	m		81.6
17	—			82.1	—			99.3	—			98.8
18	1.28	s		21.1	1.20	s		18.2	1.24	s		19.0
19	1.37	s		17.4	0.99	s		19.3	1.00	s		19.3
20	2.29	dq	11.8, 6.9	36.2	3.31	dq	10.0, 6.9	43.3	3.76	dq	8.6, 6.1	46.6
21	1.07	d	6.9	11.9	1.15	d	6.9	13.0	1.34	d	6.1	13.2
22	3.38	ddd	11.8, 10.6, 6.5	44.3	3.98	ddd	10.9, 10.0, 6.3	49.5	3.27	ddd	8.7, 8.6, 5.8	39.8
23	5.24	d	6.5	97.6	5.26	d	6.3	110.4	5.05	d	5.8	103.9
24	6.96	dd	10.6, 1.2	143.5	7.22	dd	10.9, 1.1	140.8	7.48	dd	8.7, 1.2	139.8
25	—			130.3	—			130.8	—			129.6
26	—			168.3	—			168.1	—			168.3
27	1.99	d	1.2	13.3	2.03	d	1.1	13.4	2.11	d	1.2	13.5
28	—			—	—			—	—			—
29	—			—	—			—	—			—
30	1.30	s		27.9	1.40	s		28.3	1.41	s		28.3
31	1.16	s		16.5	1.17	s		16.7	1.17	s		16.7
32	1.84	s		30.7	1.46	s		27.2	1.43	s		27.0
23-OMe	—			—	3.49	s		56.4	3.42	s		55.1
26-OMe	3.71	s		51.6	3.71	s		51.7	3.67	s		51.6

**Table 2:** ^1^H and ^13^C NMR (500 and 125 MHz, C_5_D_5_N) spectroscopic assignments of the sugar moieties of **1**–**3**.

		1				2				3			
C		δ_H_		*J* (Hz)	δ_C_	δ_H_		*J* (Hz)	δ_C_	δ_H_		*J* (Hz)	δ_C_
Glc	1′	4.91	d	7.7	105.2	4.97	d	7.6	105.2	4.97	d	8.3	105.2
	2′	4.42	dd	9.0, 7.7	78.7	4.43	dd	9.2, 7.6	79.0	4.44	dd	9.1, 8.3	79.0
	3′	4.54	t	9.0	79.5	4.56	t	9.2	79.5	4.56	t	9.1	79.5
	4′	4.06	t	9.0	71.9	4.10	t	9.2	72.0	4.10	t	9.1	71.9
	5′	3.89	ddd	9.0, 5.4, 2.7	77.9	3.94	m		77.9	3.93	m		77.9
	6′a	4.51	dd	11.9, 2.7	62.8	4.55	dd	11.7, 3.4	62.8	4.53	br d	11.4	62.8
	b	4.35	dd	11.9, 5.4		4.39	dd	11.7, 5.3		4.34	dd	11.4, 5.5	
Glc	1′′	5.83	d	6.9	101.9	5.85	d	7.5	102.0	5.86	d	7.4	102.0
	2′′	4.24	dd	8.9, 6.9	78.7	4.31	dd	8.9, 7.5	78.6	4.33	dd	8.9, 7.4	78.6
	3′′	4.23	t	8.9	79.4	4.25	t	8.9	79.4	4.25	t	8.9	79.4
	4′′	4.06	t	8.9	72.9	4.09	t	8.9	72.9	4.09	t	8.9	72.9
	5′′	3.85	ddd	8.9, 5.7, 3.0	77.6	3.85	ddd	8.9, 5.3, 2.6	77.5	3.86	ddd	8.9, 5.8, 3.1	77.5
	6′′a	4.49	dd	10.8, 3.0	63.5	4.49	dd	11.3, 2.6	63.5	4.49	dd	11.5, 3.1	63.5
	b	4.29	dd	10.8, 5.7		4.31	dd	11.3, 5.3		4.31	dd	11.5, 5.8	
Rha	1′′′	6.38	br s		102.1	6.41	br s		102.1	6.41	br s		102.1
	2′′′	4.76	br d	3.3	72.4	4.76	br d	3.3	72.4	4.76	br d	3.3	72.4
	3′′′	4.67	dd	9.3, 3.3	72.7	4.69	dd	9.2, 3.3	72.7	4.69	dd	9.3, 3.3	72.7
	4′′′	4.32	t	9.3	74.3	4.32	t	9.2	74.3	4.33	t	9.3	74.3
	5′′′	5.01	dq	9.3, 6.3	69.5	5.06	dq	9.2, 6.2	69.6	5.07	dq	9.3, 6.2	69.6
	6′′′	1.74	d	6.3	19.0	1.80	d	6.2	18.9	1.80	d	6.2	19.0

**Table 3:** ^1^H and ^13^C NMR (500 and 125 MHz, CD_3_OD) spectroscopic assignments of **12** and **13**.

		12				13			
C		δ_H_		*J* (Hz)	δ_C_	δ_H_		*J* (Hz)	δ_C_
2		4.36	dd	11.5, 4.0	70.8	4.35	dd	11.5, 4.5	71.4
		4.19	dd	11.5, 6.5		4.20	dd	11.5, 7.0	
3		2.96	m		48.2	2.96	m		49.4
4		—			200.0	—			201.7
4	a	—			101.8	—			105.2
5		—			155.7	—			156.4
6		—			131.2	—			132.0
7		—			160.8	—			160.8
8		6.31	s		97.4	6.31	s		98.1
8	a	—			160.1	—			162.2
9		3.13	dd	14.0, 9.0	33.0	3.09	dd	14.0, 9.5	33.9
		2.72	dd	14.0, 7.0		2.68	dd	14.0, 7.3	
1′		—			131.2	—			132.0
2′		7.16	d	8.5	131.2	7.06	d	8.5	132.0
3′		6.86	d	8.5	115.2	6.73	d	8.5	117.2
4′		—			160.0	—			158.3
5′		6.86	d	8.5	115.1	6.73	d	8.5	117.2
6′		7.16	d	8.5	131.2	7.06	d	8.5	132.0
6-OCH_3_		3.75	s		61.4	3.75	s		62.7
4′-OCH_3_		3.77	s		55.7	—			
Glc 1′′		4.96	d	7.5	101.7	4.95	d	7.5	102.5
2′′		3.49	dd	8.5, 7.5	74.8	3.50	dd	8.7, 7.5	75.5
3′′		3.46	t	8.5	78.1	3.46	t	8.7	78.8
4′′		3.36	dd	9.2, 8.5	71.4	3.63	t	8.7	72.2
5′′		3.59	m		77.3	3.59	m		78.1
6′′		4.02	dd	12.5, 5.0	67.6	4.01	br d	11.2	68.3
		3.59	br d	12.5		3.60	br d	11.0	
									
Rha 1′′′		4.68	br s		102.2	4.68	br s		103.0
2′′′		3.88	br s		72.1	3.88	br dd	3.4, 1.4	72.9
3′′′		3.69	dd	9.6, 3.5	72.5	3.69	dd	9.8, 3.5	73.2
4′′′		3.32	t	9.6	74.1	3.33	t	9.8	74.9
5′′′		3.62	m		69.8	3.62	m		70.6
6′′′		1.18	d	6.3	18.0	1.18	br d	6.2	15.2

**Table 4:** The pancreatic lipase inhibitory activity of **1**–**11**.

Compounds	IC_50_ (mM)
Cetilistat	1.43 ± 0.052 μM
**1**	–
**2**	0.84 ± 0.029
**3**	0.59 ± 0.020
**4**	1.42 ± 0.040
**5**	1.40 ± 0.035
**6**	0.81 ± 0.029
**7**	1.10 ± 0.015
**8**	-
**9**	0.91 ± 0.023
**10**	1.13 ± 0.062
**11**	>4.50

Data are represented as the mean ± S.E.M. of three experiments performed in triplicate.

## Data Availability

Data is contained within the article or supplementary material.

## Supplementary Materials

The supplementary materials can be found online at https://www.mdpi.com/1422-0067/22/3/1262/s1.
