# Supplementary material for: Chemical Constituents of the Bulbs of Scilla peruviana and Their Pancreatic Lipase Inhibitory Activity
Source: Int J Mol Sci. 2021 Jan 27;22(3):1262. doi: 10.3390/ijms22031262 (PMC7865276; doi:10.3390/ijms22031262)
Supplement: Supplementary file 1 [file ijms-22-01262-s001.pdf]

Supplementary Materials

Article

Chemical constituents of the bulbs of *Scilla peruviana* and their pancreatic lipase inhibitory activity

Yukiko Matsuo *, Asuka Yamashiro , Kanae Ootomo , Mika Nakagawa , Hiroko Tsuchihashi , Niro Inaba , and Yoshihiro Mimaki

School of Pharmacy, Tokyo University of Pharmacy and Life Sciences, 1432-1, Horinouchi, Hachioji, Tokyo 192-0392, Japan.; y104219@toyaku.ac.jp(A.Y.); y114037@toyaku.ac.jp(K.O.); y134172@toyaku.ac.jp(M.N.); 154150@toyaku.ac.jp(H.T.); ninaba@toyaku.ac.jp(N.I.); mimakiy@toyaku.ac.jp (Y.M.)

* Correspondence: matsuoy@toyaku.ac.jp; Tel.: +81-42-676-4577

Figures

Page

S.1. Compound **1**
^{1}H NMR, ^{13}C NMR, DEPT, HMBC, HMQC, NOESY, UV, IR, and MS
………………….page 3

S.2. Compound **2**
^{1}H NMR, ^{13}C NMR, DEPT, HMBC, HMQC, NOESY, UV, IR, and MS
………………….page 6

S.3. Compound **3**
^{1}H NMR, ^{13}C NMR, DEPT, HMBC, HMQC, NOESY, UV, IR, and MS
………………….page 10

S.4. Compound **12**
^{1}H NMR, ^{13}C NMR, DEPT, HMBC, HMQC, NOESY, UV, IR, and MS
………………….page 14

S.5. Compound **13**
^{1}H NMR, ^{13}C NMR, DEPT, HMBC, HMQC, NOESY, UV, IR, and MS
………………….page 17

S.6. The purities of test samples (**1-11**) calculated by HPLC analysis
………………….page 21

S.1. Compound 1 ^{1}H NMR (500 MHz, pyridine-d_5) spectrum of **1**

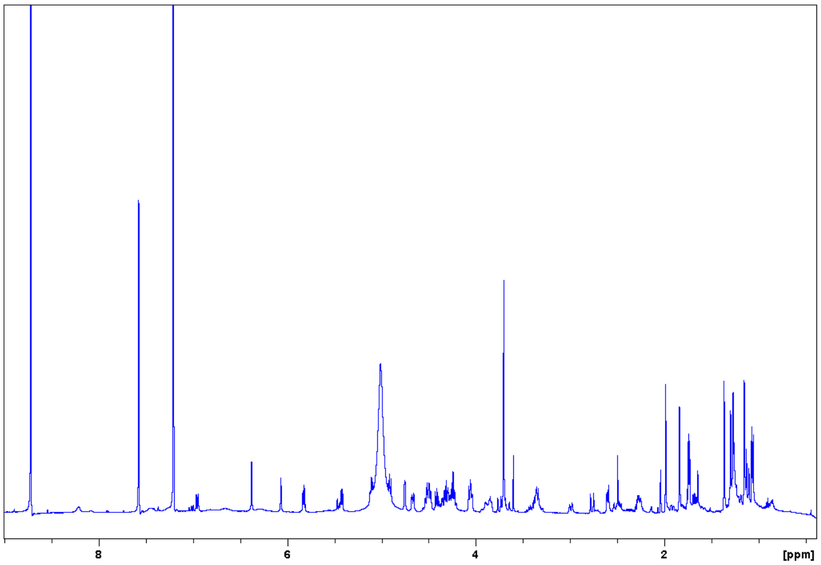

^{13}C NMR (125 MHz, pyridine-d_5) spectrum of **1**

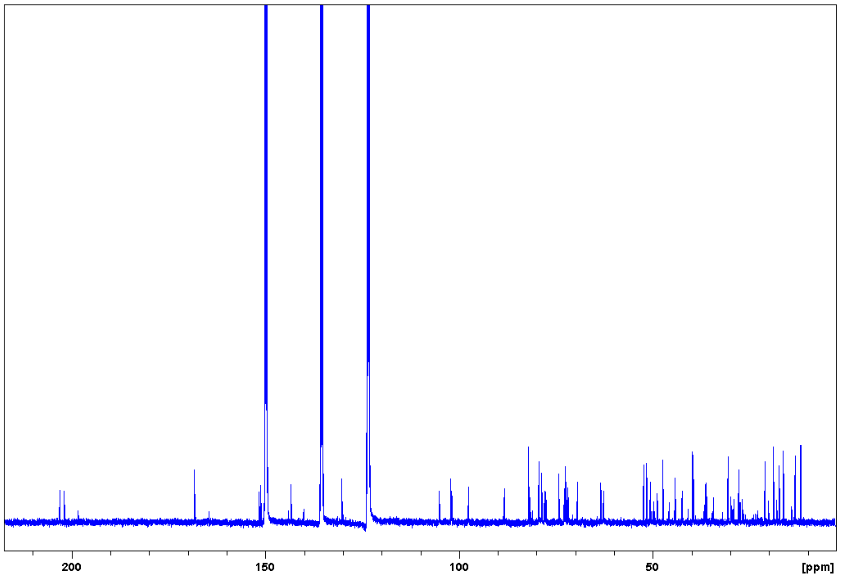

DEPT spectrum of **1**

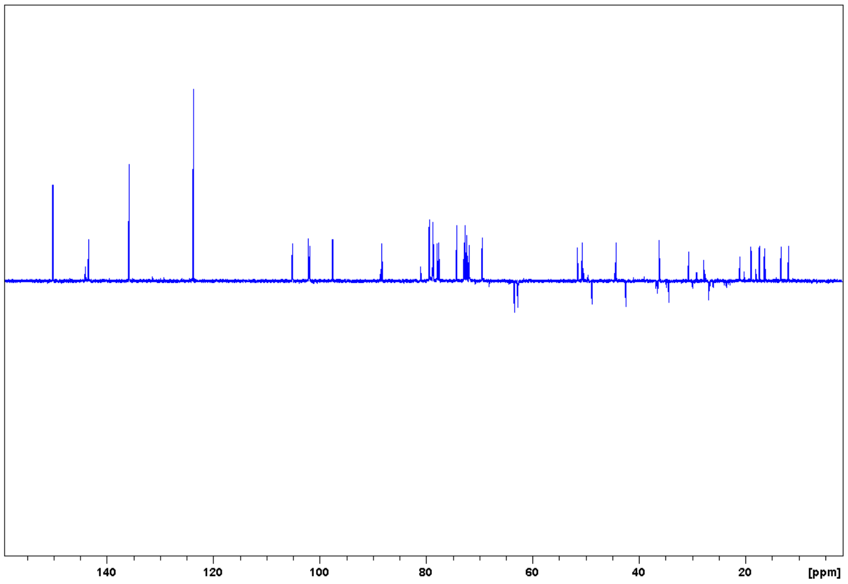

HMBC spectrum of **1**

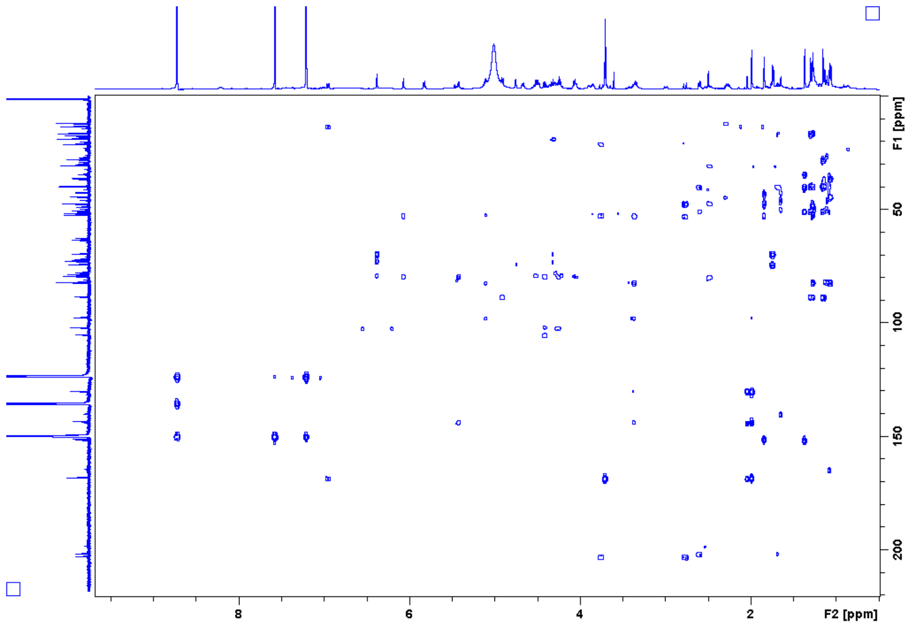

HMQC spectrum of **1**

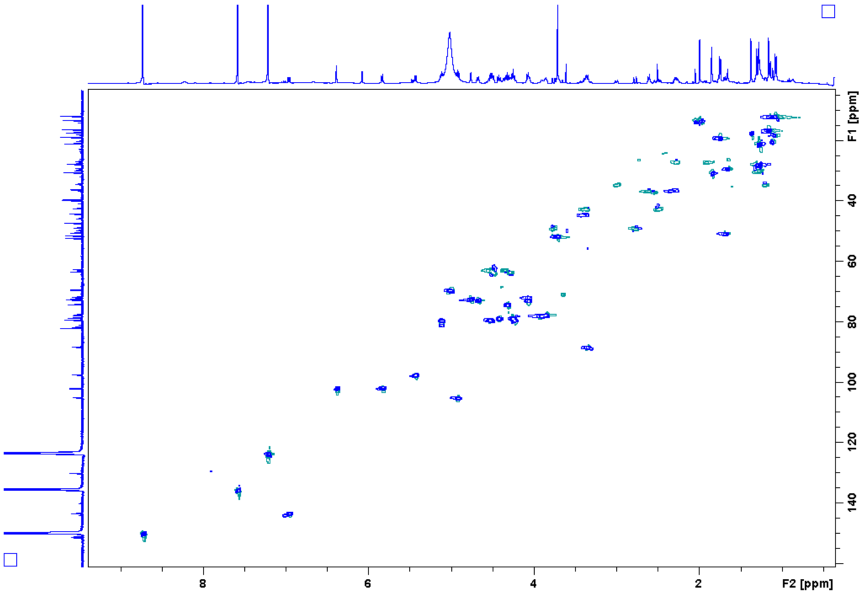

NOESY spectrum of **1**

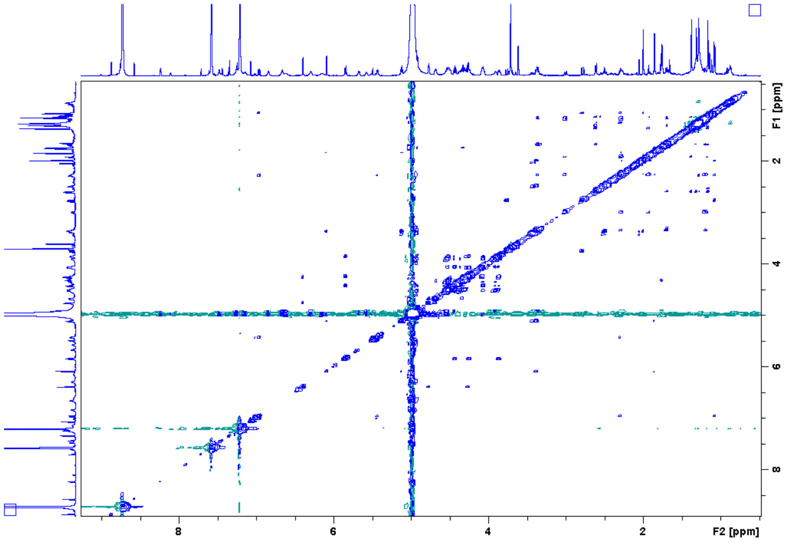

UV, IR, and MS of **1**

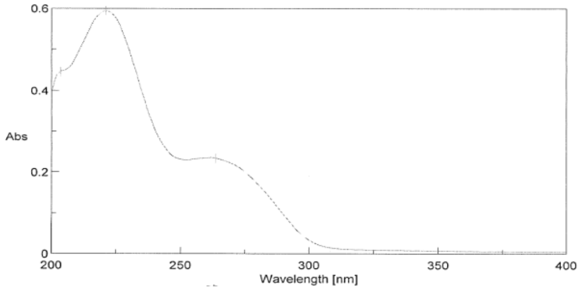

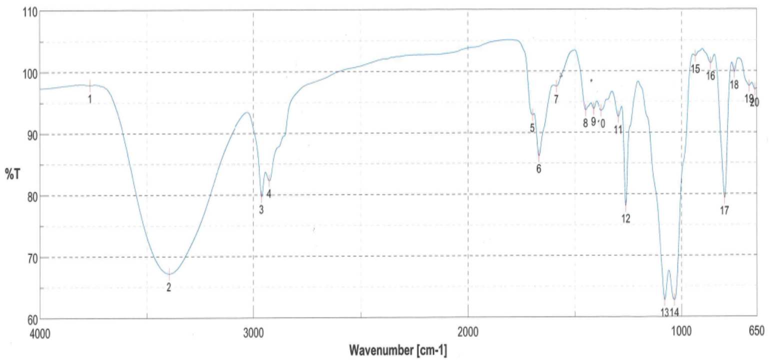

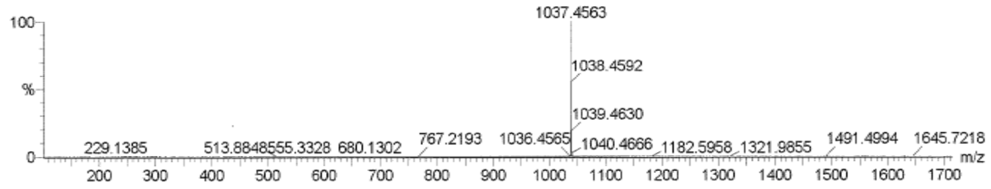

Minimum:				-1.5				
Maximum:		10.0	20.0	300.0				
Mass	Calc. Mass	mDa	PPM	DBE	i-FIT	Norm	Conf(%)	Formula
1037.4563	1037.4569	-0.6	-0.6	12.5	232.7	n/a	n/a	C49 H74 O22 Na

S.2. Compound 2 ^{1}H NMR (500 MHz, pyridine-d_5) spectrum of **2**

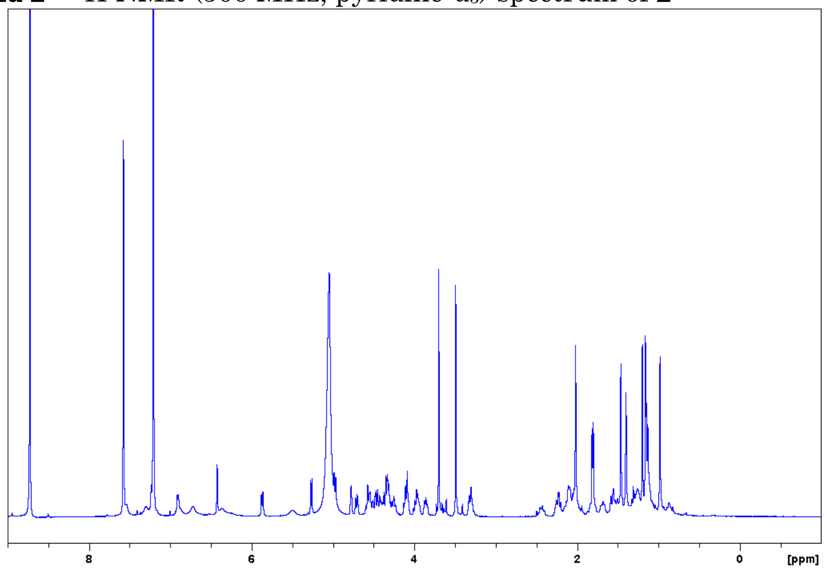

^{13}C NMR (125 MHz, pyridine-d_5) spectrum of **2**

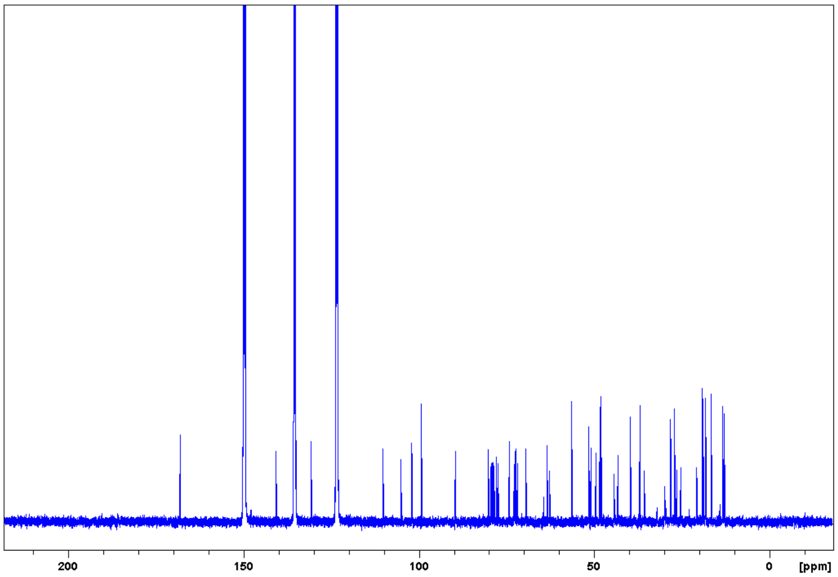

DEPT spectrum of **2**

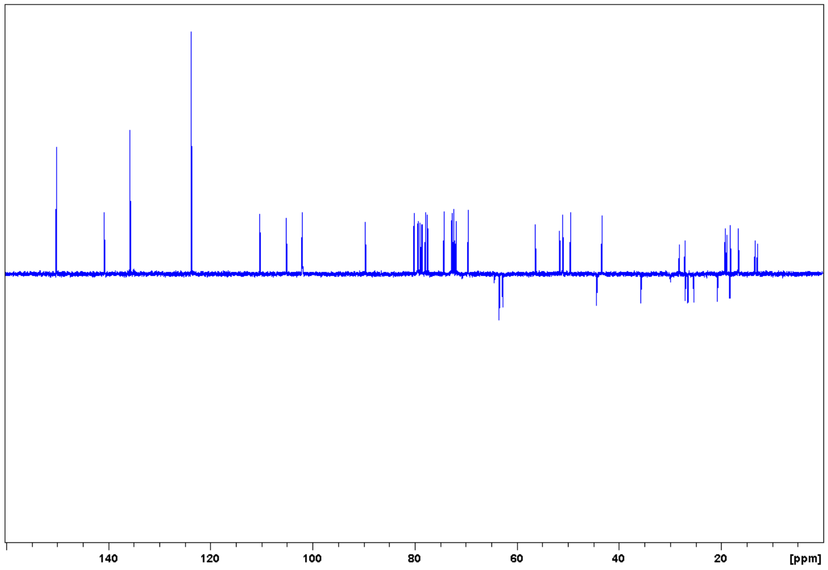

HMBC spectrum of **2**

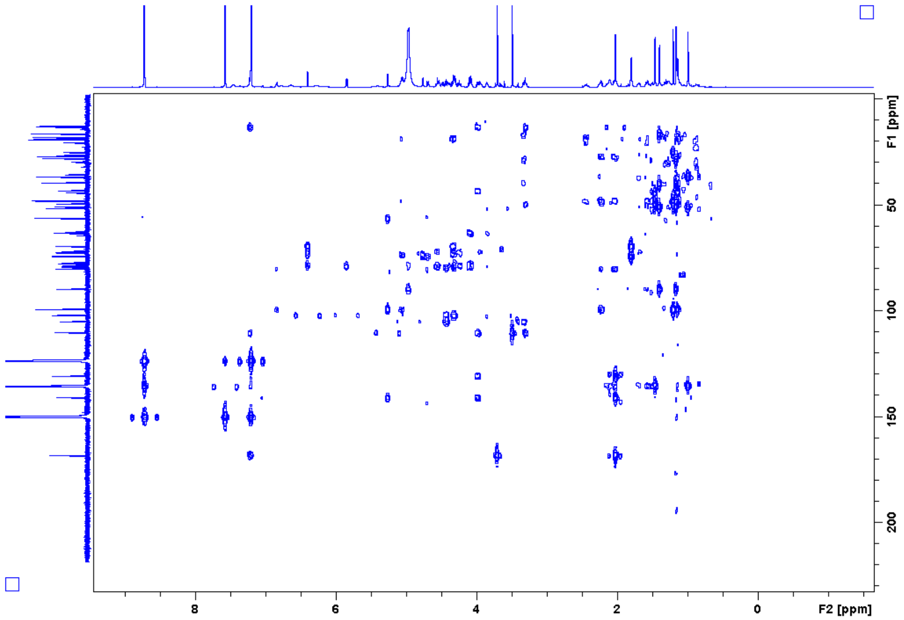

HMQC spectrum of **2**

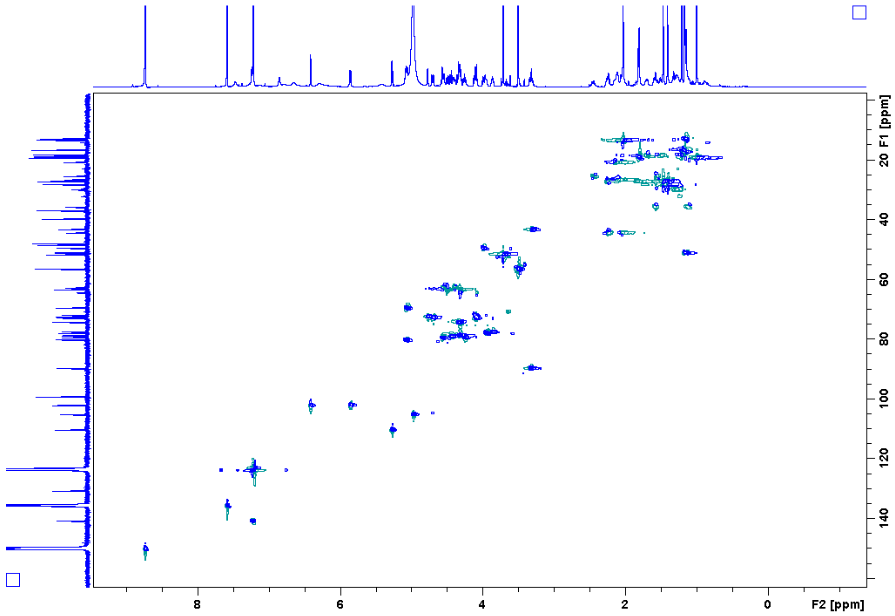

NOESY spectrum of **2**

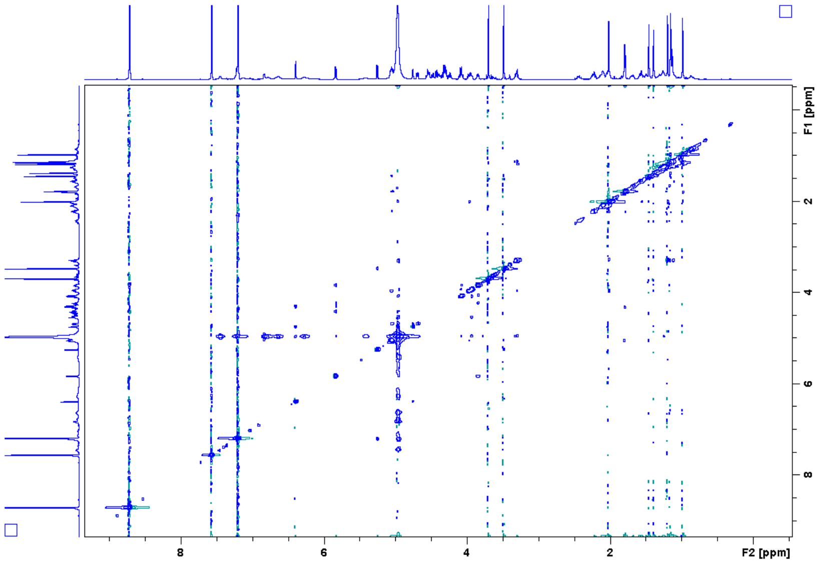

UV, IR, and MS of **2**

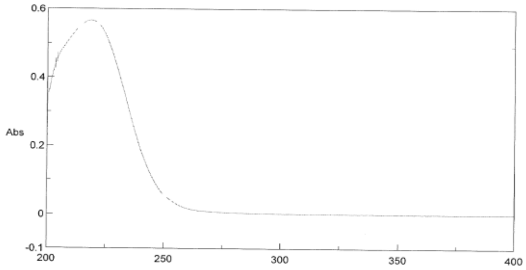

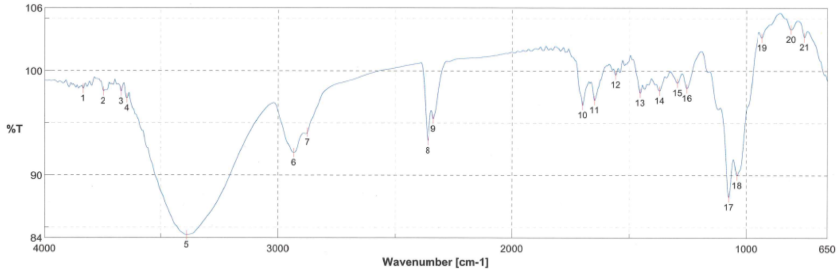

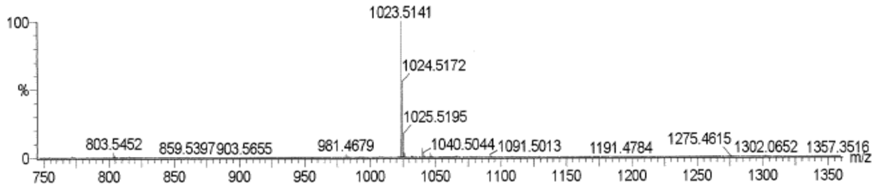

	Mass	Calc. Mass	mDa	PPM	DBE	i-FIT	Norm	Conf(%)	Formula
Minimum:					-1.5				
Maximum:			10.0	20.0	300.0				
	1023.5141	1023.5141	0.0	0.0	10.5	308.1	n/a	n/a	C50 H80 O20 Na

S.3. Compound 3 ^{1}H NMR (500 MHz, pyridine-d_5) spectrum of **3**

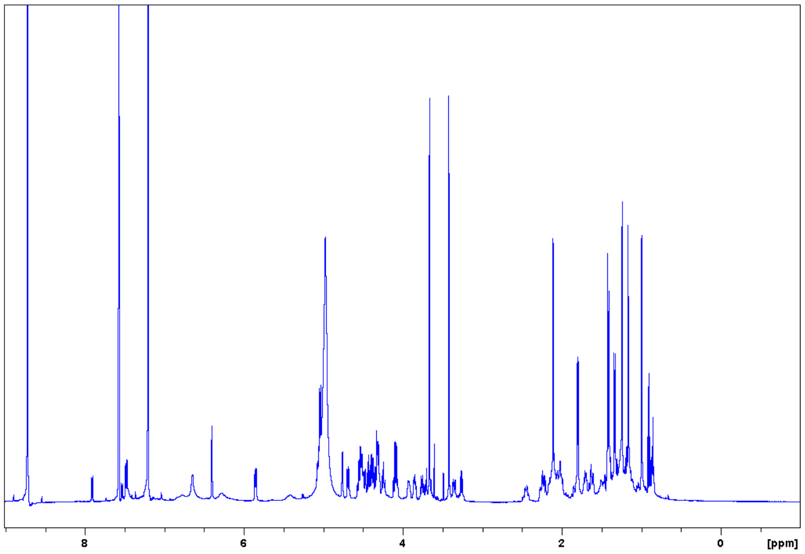

^{13}C NMR (125 MHz, pyridine-d_5) spectrum of **3**

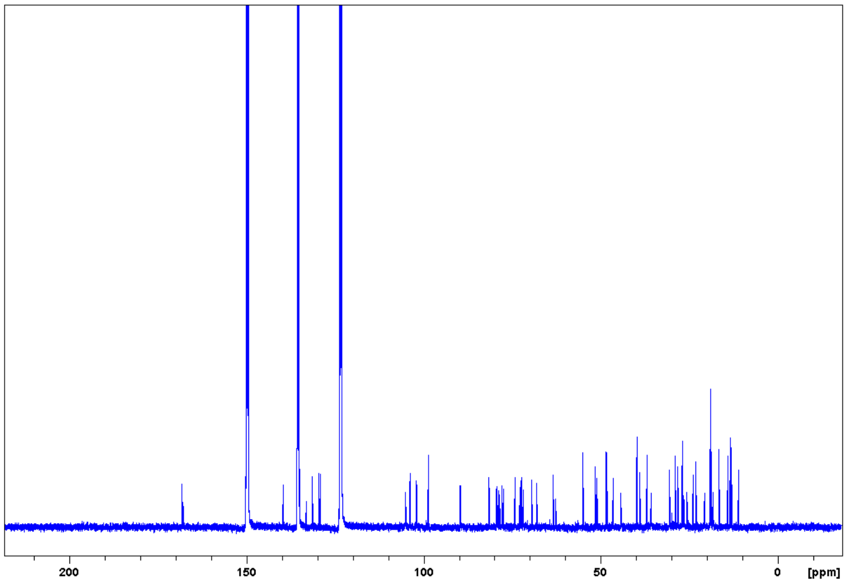

DEPT spectrum of **3**,

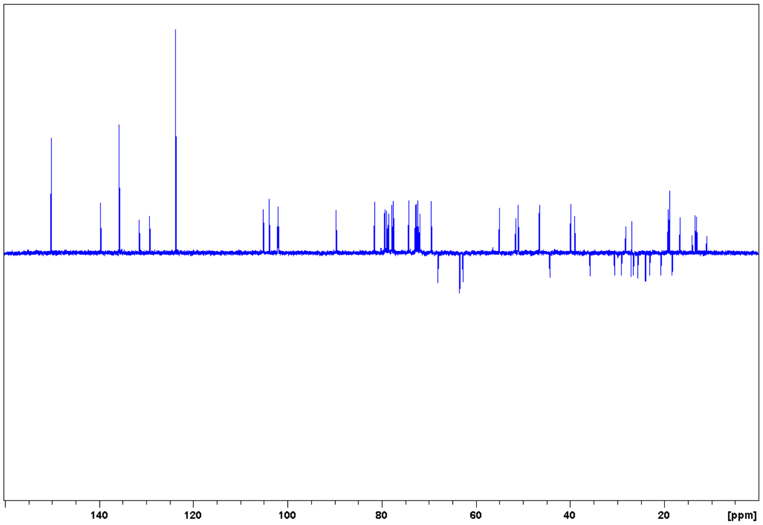

HMBC spectrum of **3**

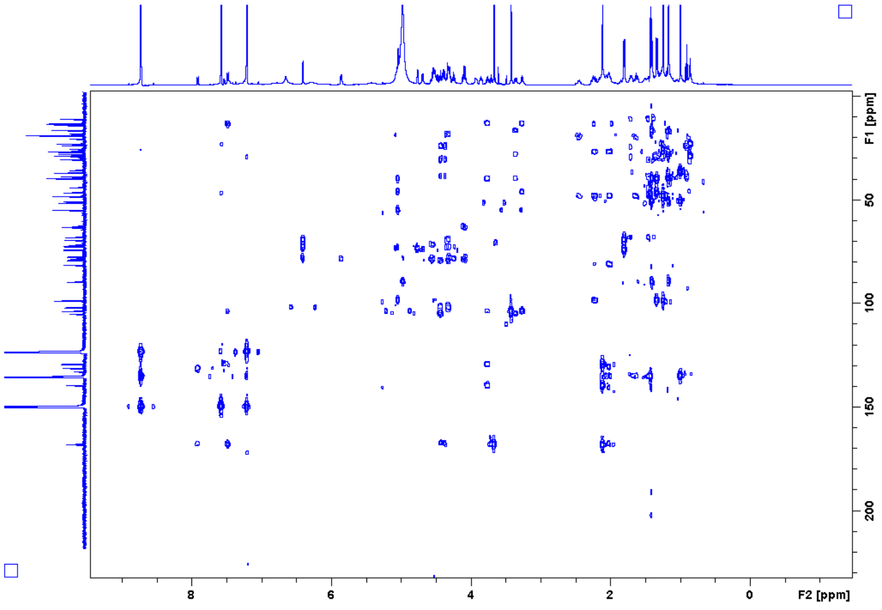

HMQC spectrum f **3**

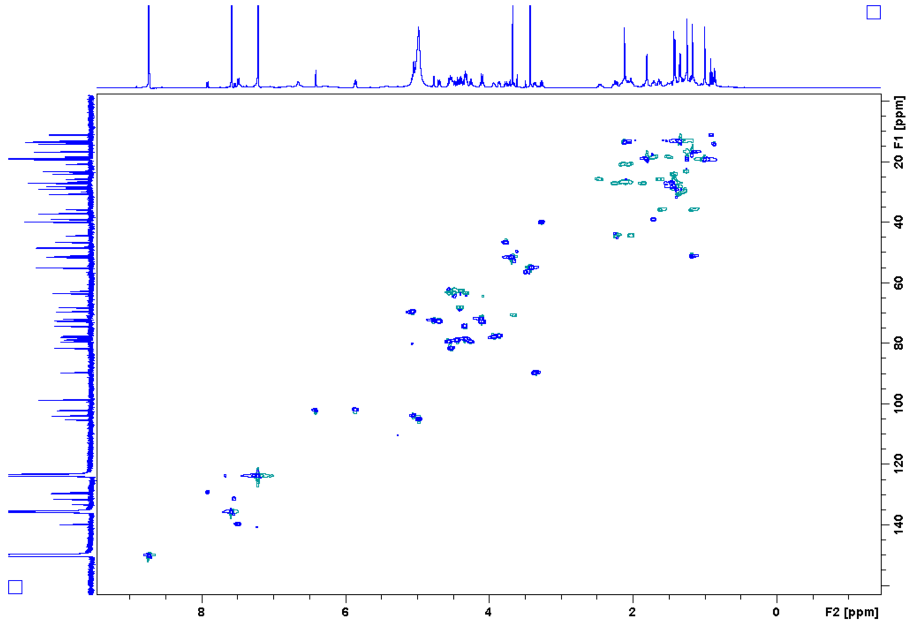

NOESY spectrum of **3**,

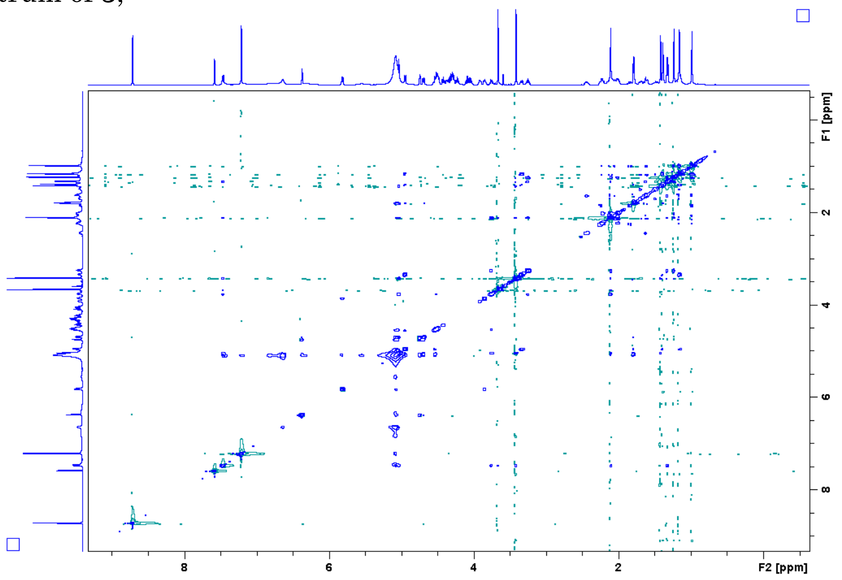

UV, IR, and MS of **3**

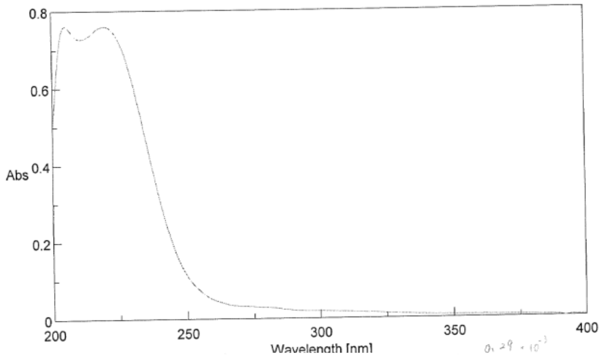

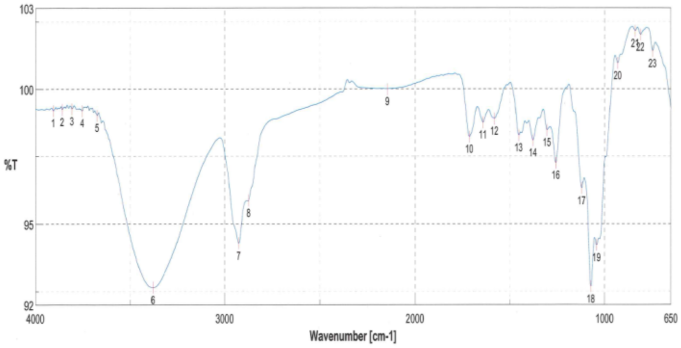

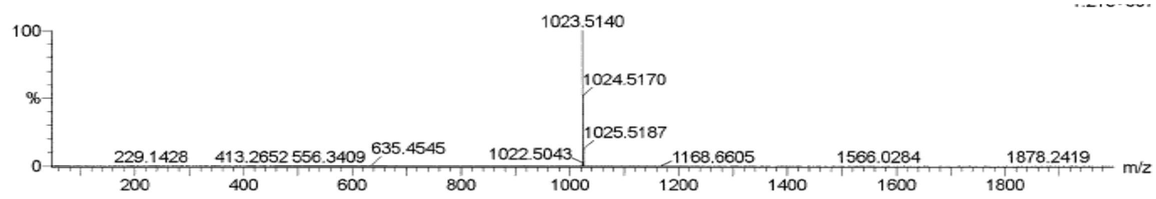

Minimum:				-1.5				
Maximum:		10.0	20.0	300.0				
Mass	Calc. Mass	mDa	PPM	DBE	i-FIT	Norm	Conf(%)	Formula
1023.5140	1023.5141	-0.1	-0.1	10.5	279.9	n/a	n/a	C50 H80 O20 Na

S.4. Compound 12 ^{1}H NMR (500 MHz, methanol-d_5) spectrum of **12**

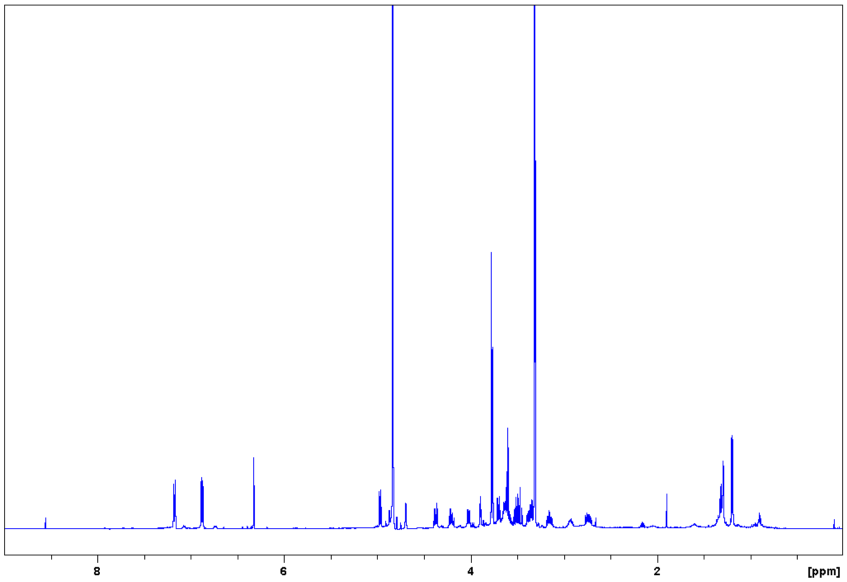

^{13}C NMR (125 MHz, methanol-d_5) spectrum of **12**

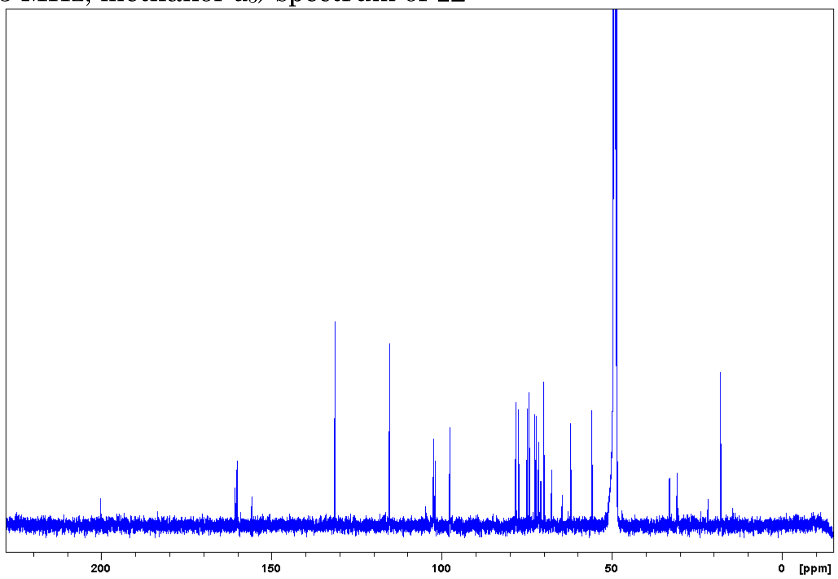

DEPT spectrum of **12**

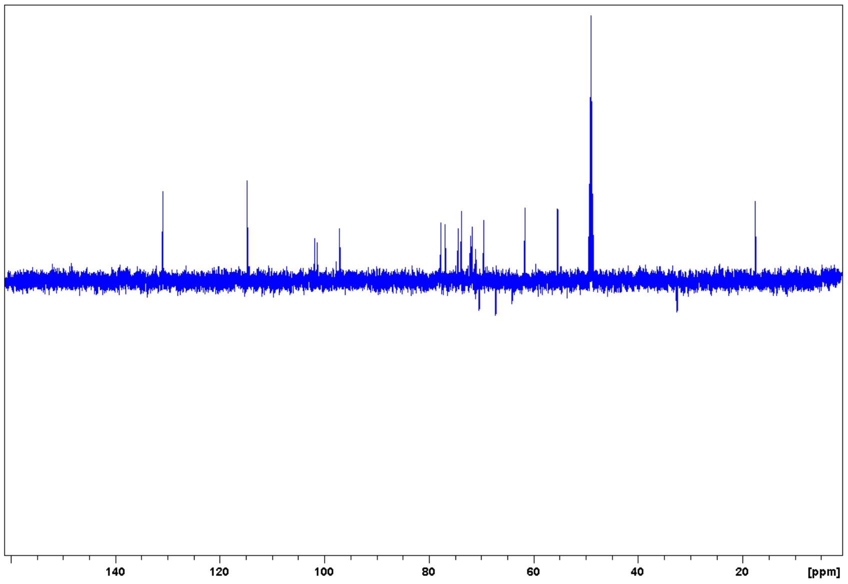

HMBC spectrum of **12**

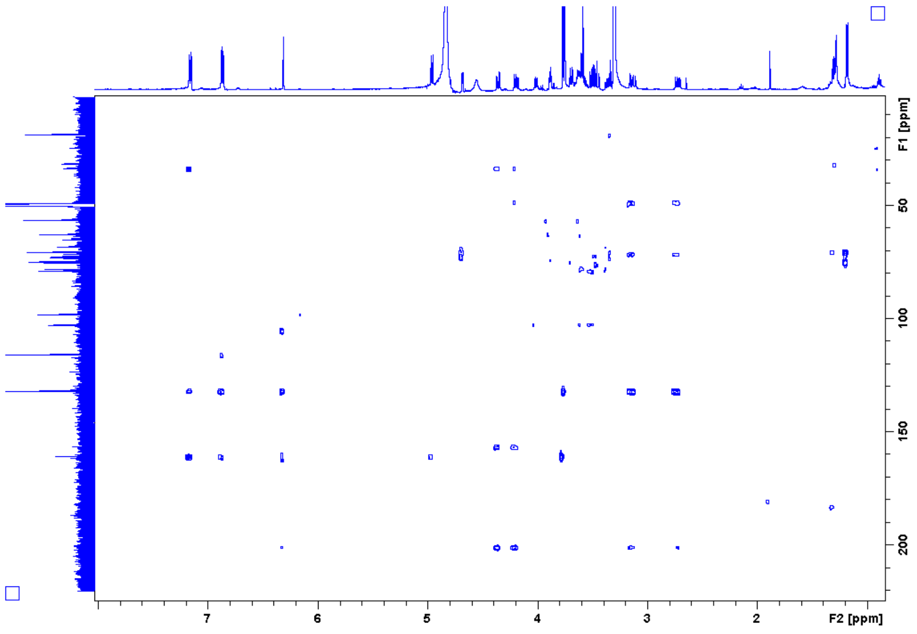

HMQC spectrum of **12**

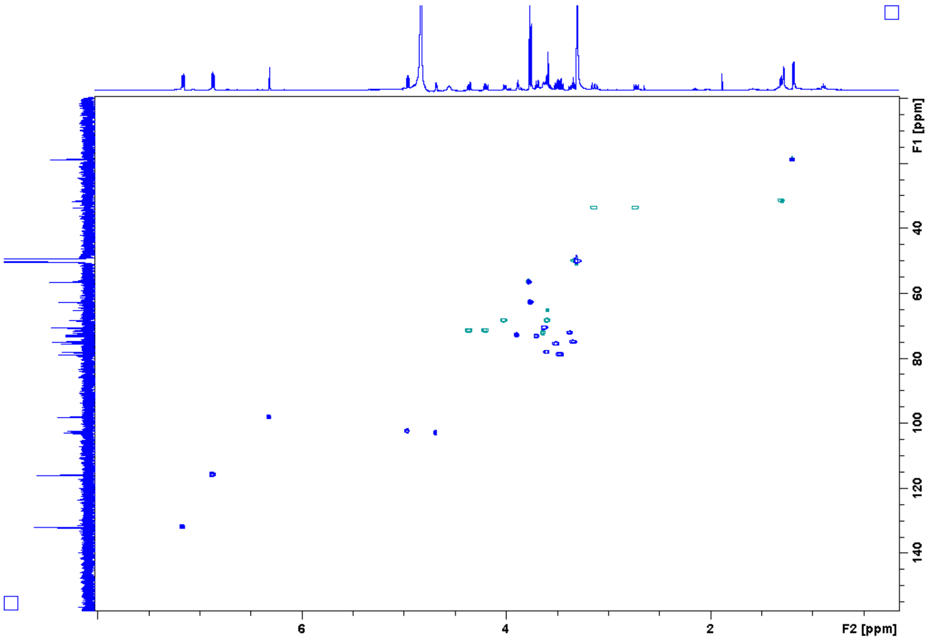

UV, IR, and MS of **12**

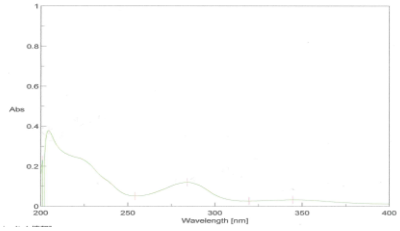

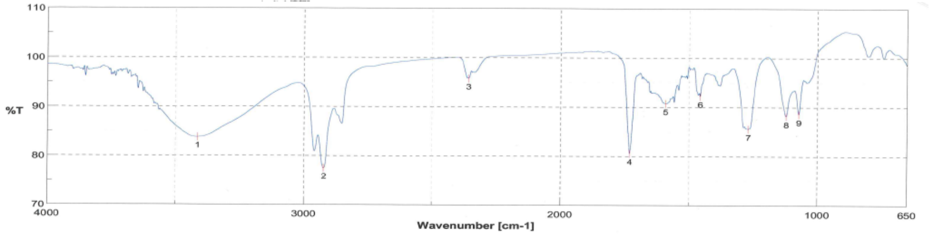

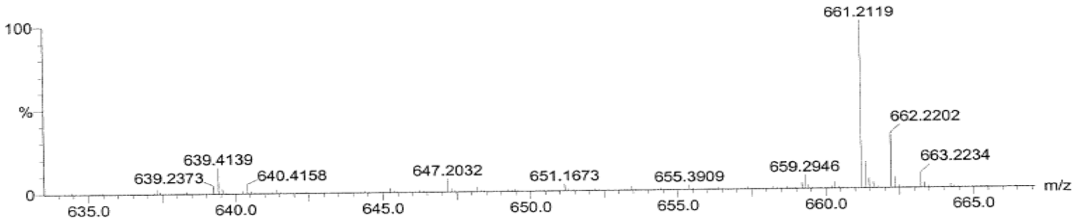

Minimum:				0.5		
Maximum:		50.0	20.0	500.0		
Mass	Calc. Mass	mDa	PPM	DBE	i-FIT	Formula
661.2119	661.2108	1.1	1.7	11.5	18.9	C30 H38 O15 Na

S.5. Compound 13 ^{1}H NMR (500 MHz, methanol-d_5) spectrum of **13**

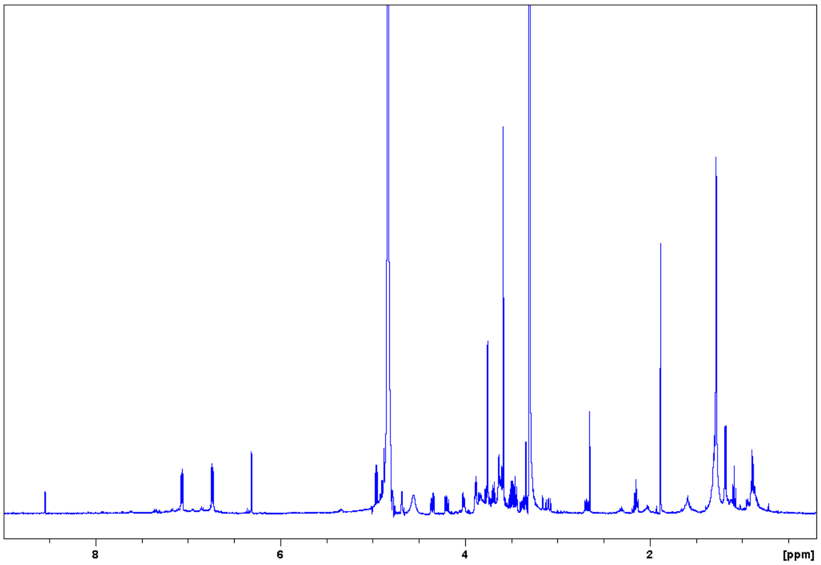

^{13}C NMR (125 MHz, methanol-d_5) spectrum of **13**

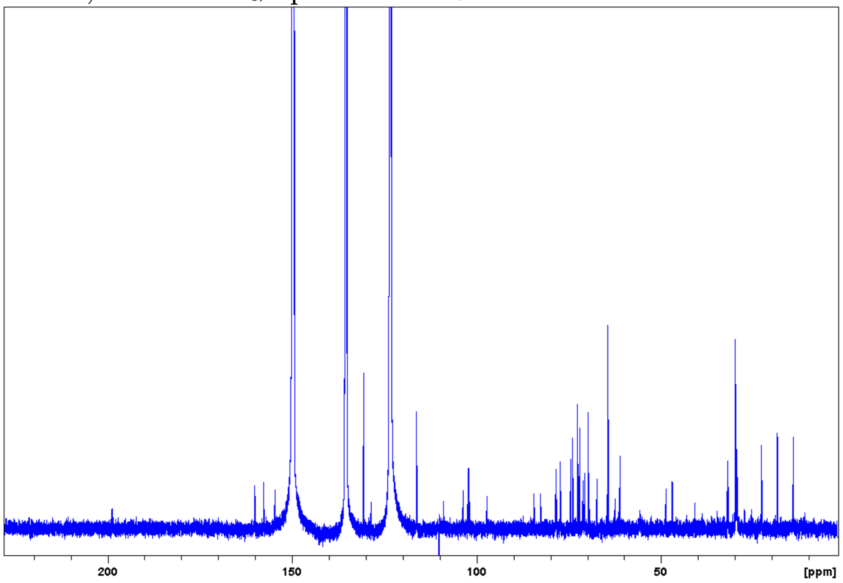

DEPT spectrum of **13**

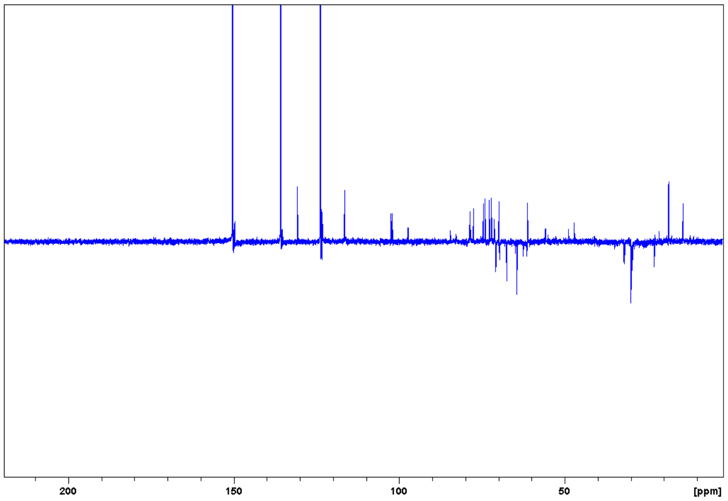

HMBC spectrum of **13**

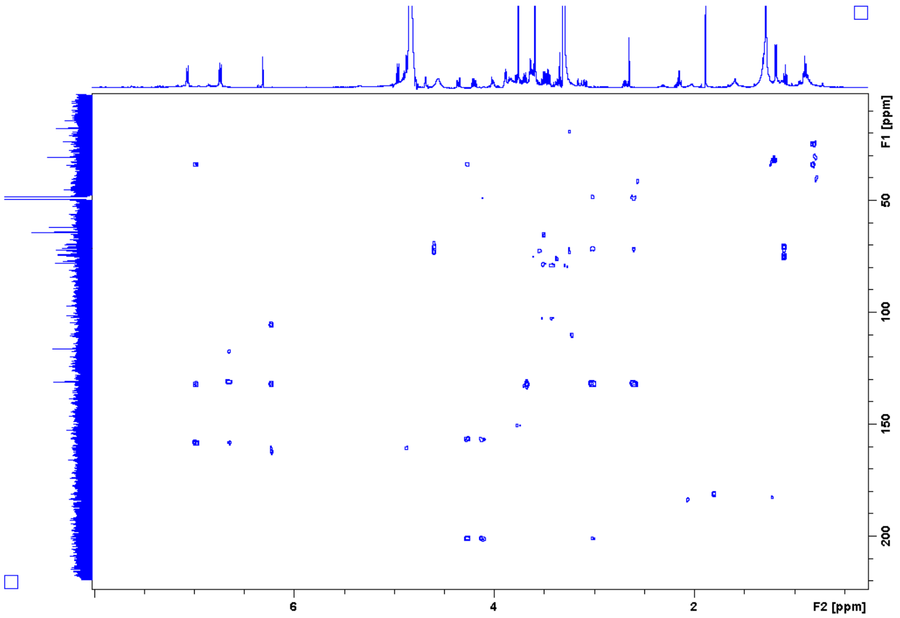

HMQC spectrum of **13**

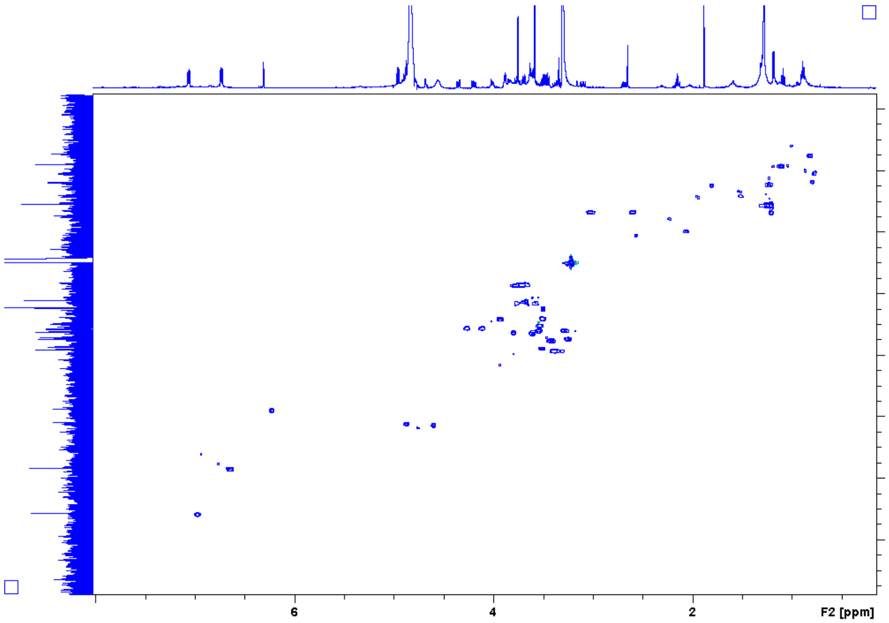

UV, IR, and MS of **13**

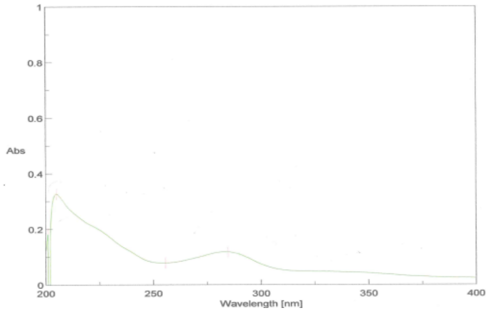

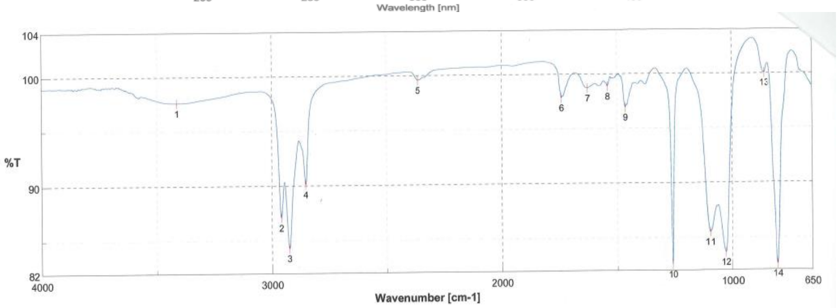

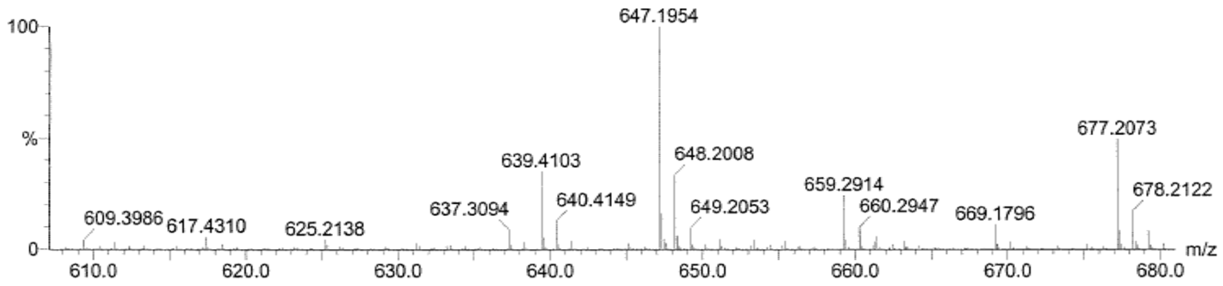

Minimum:				0.5		
Maximum:		100.0	50.0	500.0		
Mass	Calc. Mass	mDa	PPM	DBE	i-FIT	Formula
647.1954	647.1952	0.2	0.3	11.5	29.6	C29 H36 O15 Na

S.6. The purities of test samples (1-11) calculated by HPLC analysis

HPLC was conducted using a system consisting of a CCPM pump (Shimadzu), an RI-8021 (Tosoh) detector, and a Rheodyne injection port (Rohnert Park). A TSK gel ODS-100Z column (Tosoh) was used for the HPLC.

Solvent **1**: MeOH-H_2O 4:1; **2**: MeOH-H_2O 6:1; **3**: MeOH-H_2O 6:1; **4**: MeOH-H_2O 3:1; **5**: MeOH-H_2O 3:1; **6**: MeOH-H_2O 6:1; **7**: MeOH-H_2O 6:1; **8**: MeOH-H_2O 6:1; **9**: MeOH-H_2O 6:1; **10**: MeOH-H_2O 6:1; **11**: MeOH-H_2O 4:1

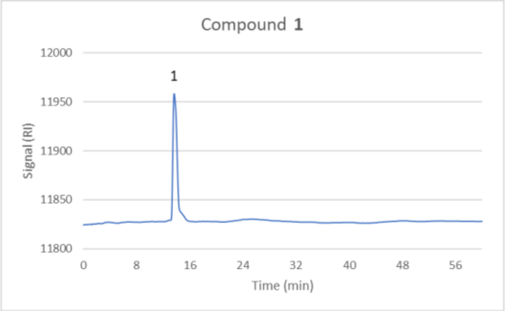

No	Rt(min)	Area(%)
1	13.68	100

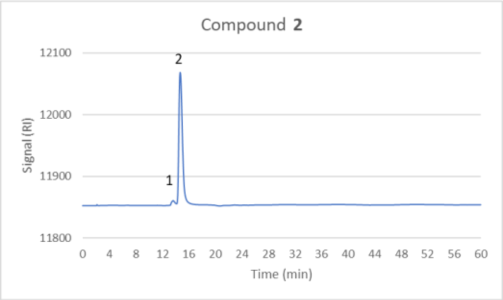

No	Rt(min)	Area(%)
1	13.65	1.2
2	14.71	98.8

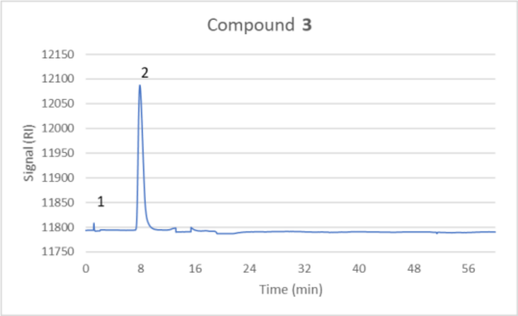

No	Rt(min)	Area(%)
1	1.18	0.4
2	7.92	99.6

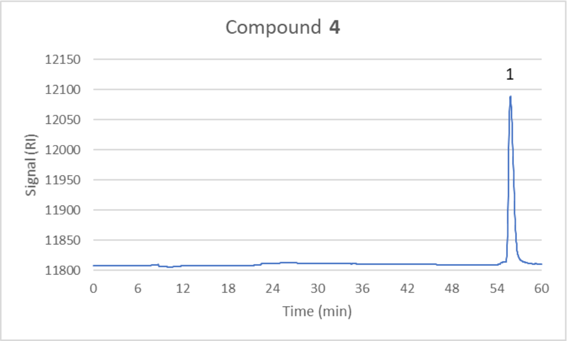

No	Rt(min)	Area(%)
1	55.86	100

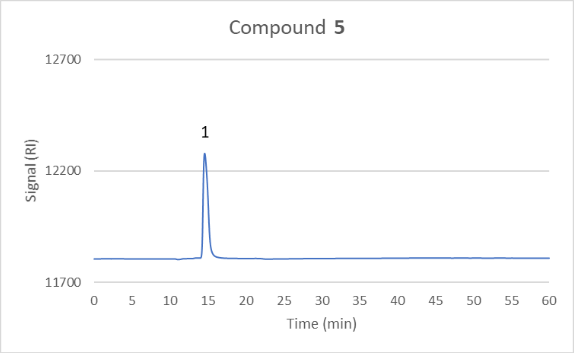

No	Rt(min)	Area(%)
1	14.57	100

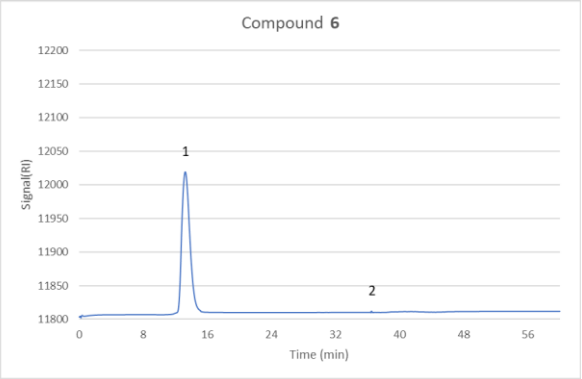

No	Rt(min)	Area(%)
1	13.23	99.7
2	36.50	0.3

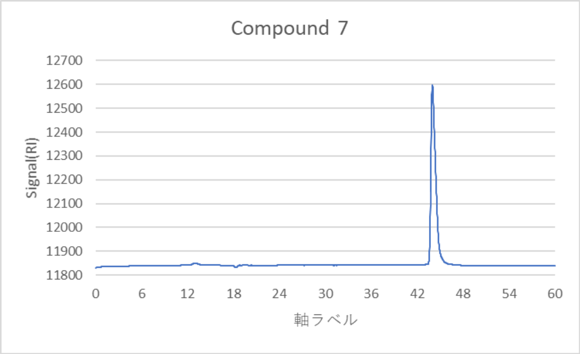

No	Rt(min)	Area(%)
1	43.99	100

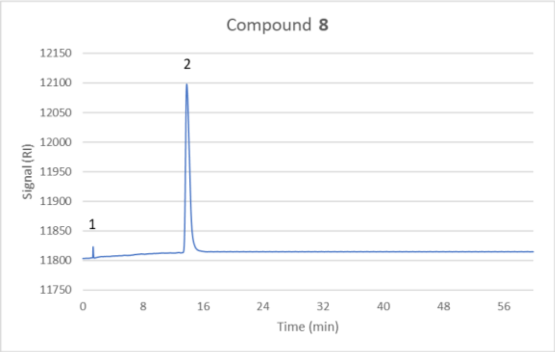

No	Rt(min)	Area(%)
1	1.37	0.7
2	13.84	99.3

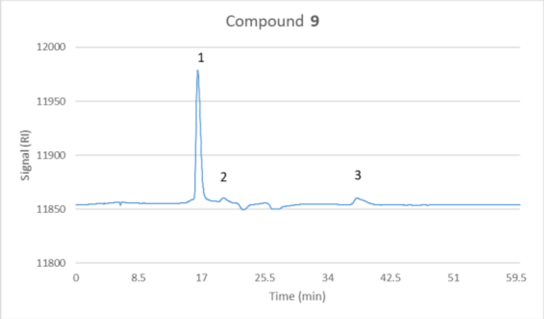

No	Rt(min)	Area(%)
1	16.47	98.4
2	17.91	1.1
3	38.02	0.5

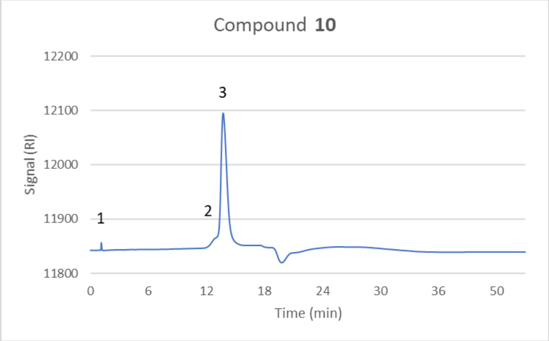

No	Rt(min)	Area(%)
1	1.12	0.5
2	13.12	0.5
3	13.73	99

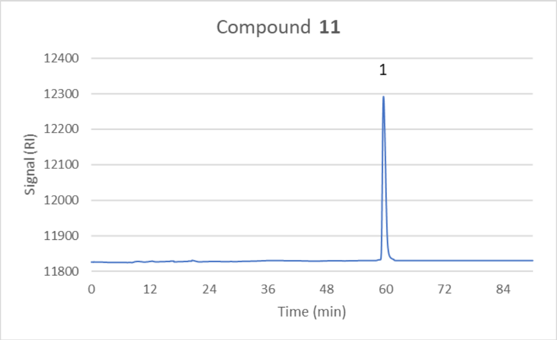

No	Rt(min)	Area(%)
1	59.58	100
